# SEGCECO: Subgraph Embedding of Gene expression matrix for prediction of CEll-cell COmmunication

**DOI:** 10.1093/bib/bbae160

**Published:** 2024-04-11

**Authors:** Akram Vasighizaker, Sheena Hora, Raymond Zeng, Luis Rueda

**Affiliations:** School of Computer Science, University of Windsor, Windsor, ON, Canada; Software Development Department, Amazon, USA; School of Computer Science, University of Windsor, Windsor, ON, Canada; School of Computer Science, University of Windsor, Windsor, ON, Canada

**Keywords:** single-cell transcriptomic, cell–cell interaction, link prediction, graph convolutional network, sub-graph embedding

## Abstract

Recent advances in single-cell RNA sequencing technology have eased analyses of signaling networks of cells. Recently, cell–cell interaction has been studied based on various link prediction approaches on graph-structured data. These approaches have assumptions about the likelihood of node interaction, thus showing high performance for only some specific networks. Subgraph-based methods have solved this problem and outperformed other approaches by extracting local subgraphs from a given network. In this work, we present a novel method, called Subgraph Embedding of Gene expression matrix for prediction of CEll-cell COmmunication (SEGCECO), which uses an attributed graph convolutional neural network to predict cell–cell communication from single-cell RNA-seq data. SEGCECO captures the latent and explicit attributes of undirected, attributed graphs constructed from the gene expression profile of individual cells. High-dimensional and sparse single-cell RNA-seq data make converting the data into a graphical format a daunting task. We successfully overcome this limitation by applying SoptSC, a similarity-based optimization method in which the cell–cell communication network is built using a cell–cell similarity matrix which is learned from gene expression data. We performed experiments on six datasets extracted from the human and mouse pancreas tissue. Our comparative analysis shows that SEGCECO outperforms latent feature-based approaches, and the state-of-the-art method for link prediction, WLNM, with 0.99 ROC and 99% prediction accuracy. The datasets can be found at https://www.ncbi.nlm.nih.gov/geo/query/acc.cgi?acc=GSE84133 and the code is publicly available at Github https://github.com/sheenahora/SEGCECO and Code Ocean https://codeocean.com/capsule/8244724/tree.

## INTRODUCTION

In the graph domain, link prediction is the problem of predicting the existence of a connection between two entities in a network. Given a network with various nodes connected, we want to predict if two nodes are connected or are likely to connect in the future. With graph neural networks (GNN), we use not only network structural information, such as connections between nodes but also individual node characteristics including the feature set of the node. Predicting friendship links among users in a social network, predicting co-authorship links in a citation network, and predicting interactions between genes and proteins in a biological network are some examples of link prediction. Cell–cell interactions regulate organism development by cell functions. A disease may occur when cells do not interact properly or decode molecular messages improperly. Thus, identifying and quantifying signalling interactions between cells has become a common analysis carried out across a variety of fields [1].

With the rapid advancement of single-cell RNA sequencing (scRNA-seq) technologies, researchers are becoming more interested in inferring cell–cell communication (CCC) from scRNA-seq data. There are a variety of computational tools and resources including ProximID [2], CellChat [3], CellTalker [4], iTalk [5], SingleCellSignalR [6], CellPhoneDB [7], SpotSC [8] and sensor [9], among others, which are available to predict CCC using gene expression profile obtained from scRNA-seq data. The resulting predictions generate testable hypotheses about cell–cell interactions that can be validated more thoroughly through experimental methods. Generally, in scRNA-seq data analysis, cells are clustered based on their gene expression profiles, and cell types are determined and assigned to clusters based on the known marker genes. Some CCC tools predict extracellular (i.e. interactions of genes or proteins in different cells vs intracellular, which means interactions inside a single individual cell) interactions based on ligand-receptor (LR) interactions between pairs of clusters, i.e. cell types, in which one cluster is the source and the other is the target. The majority of the tools are made up of two main components: (1) a prior knowledge resource of extracellular interactions (LR interactions) and (2) a method for estimating CCC based on known interactions and the present dataset. Each tool uses different methods, such as permutation of cluster labels, differential combinations, regularizations and scaling, depending on the input datasets. These approaches result in a varied scoring system, which makes it difficult to compare and evaluate the performance of CCC methods. Thus, selecting the appropriate tool to produce the best results is challenging [10]. A recent review study [1] discusses several existing tools for measuring CCC.

In this work, to predict CCC, we resort to various approaches that have been successfully used for other existing link prediction problems, such as the prediction of social connections between users in social networks [11]. Traditional approaches include heuristic methods such as common neighbours (CN) [12], Adamic Adar [13] and Resource Allocation) [14]. Heuristic link prediction methods use network structure, i.e. network topology information, in the prediction process. Existing algorithms can be classified based on the maximum hop of neighbours required to calculate the score [15]. CN, for example, are first-order heuristics that involve the target nodes’ one-hop neighbours. Also, some supervised approaches are used for connection prediction, including support vector machine, baggings and naive Bayes, which are used to model the problem as a binary classification in which extraction of edge features is fundamental. Moreover, recent methods are mostly built on top of node embedding methods (e.g. DeepWalk [16], node2vec [17] and structural deep network embedding [18]), with the edge representation constructed from the interaction between corresponding node embeddings. We discovered that some methods perform well on certain types of networks. For instance, every heuristic technique is based on some assumptions and works based on the extracted pattern from the network topology, which is why there is no single heuristic method that works well for all types of networks. For example, the common neighbor heuristic operates under the assumption that if two nodes have a lot of shared neighbors, then it is probable that they will form a connection with each other. While the assumption of the common neighbor heuristic may hold in social networks, it has been proven to be incorrect in protein–protein interaction (PPI) networks. In fact, in PPI networks, two proteins having many shared neighbors decrease the likelihood of them interacting with each other. Thus, this is a significant drawback in heuristic approaches. The same can be said about latent approaches, which achieve high accuracy in some types of networks but low accuracy in others. Thus, deciding on the best link prediction approach is usually a trial-and-error process. On the other hand, the Weisfeiler–Lehman Neural Machine (WLNM) [19] is considered a state-of-the-art link prediction method based on its performance. It is a new approach based on the subgraph extraction around both source and target nodes $u$ and $v$. The local enclosing subgraph for a node pair ($u$, $v$) is the subgraph induced from the network by the union of $u$ and $v$’s neighbours up to $h$ hops. The hop is the maximum distance that node features can travel. This approach gives higher accuracy than heuristic and latent methods but requires additional computation time and memory. In addition, SEAL (Learning from Subgraphs, Embeddings, and Attributes for Link Prediction) [15] is also a subgraph method that addresses several weaknesses that WLNM has. To begin with, it enables learning not only from subgraph structures but also from latent and explicit node attributes, allowing it to incorporate a variety of information. Secondly, the fully connected neural network in WLNM is replaced by a GNN that enables graph feature learning improvement. SEAL derived $\gamma $ decaying theory and proved that a small number of hops is enough to extract high-order heuristics and outperform WLNM. As a result, we choose SEAL as the baseline for predicting links between cells in our proposed framework. It is a novel method that predicts CCC in scRNA-seq data via a gene expression attributed graph convolutional network. To our knowledge, this is the first time that an attributed graph convolutional neural network method has been used for the prediction of CCC.

Also, to obtain more precise results, nodes in cell–cell communication networks (CCN), constructed based on the SoptSc approach, represent the cells instead of groups of cells, i.e. cell types. Thus, the edges denote the connections (LR interactions) between individual cells. The method presented involves predicting cell–cell interactions by utilizing a probability model based on the expression of ligands and receptors. Inspired by SoptSC, we employ a cell–cell similarity matrix derived from gene-cell data as the input for the prediction model. SoptSC first identifies a set of LR pairs that are relevant to the data. For example, in the case of hematopoiesis, SoptSC uses a list of 144 LR pairs that are known to be involved in blood cell development. SoptSC then computes the probability of each cell expressing a ligand or a receptor, based on the normalized expression level of the corresponding gene. SoptSC then calculates the probability of similarity of each cell with another cell via (LR) pairs, based on the product of the probabilities of each cell expressing the ligand and the receptor. SoptSC finally constructs the similarity matrix $S$ by summing up the similarity probabilities of cell$_{i}$ and cell$_{j}$ overall (LR) pairs. The resulting weighted graph represents a reference/known signaling network, where the probabilities indicate the likelihood of a signal passing from one cell to another. While not fully defining interactions, these probabilities could serve as necessary conditions for further predictions based on the known data.

Our study aims to predict cell interactions, with nodes representing cells in the CCN and edges representing cell–cell interactions. Thus, we use similarity matrix-based optimization for scRNA-seq data analysis tool (SpotSC) [8] to perform such a task. Once the CCN network is constructed, our main goal is to predict links among the cells. The main contributions of this paper are highlighted as follows:

- We propose a new pipeline for cell–cell interaction prediction in the scRNA-seq data using state-of-the-art studies in link prediction.
- We introduce a statistically significant pooling layer that employs information gain as an approach for coarsening graph attributes from the scRNA-seq data while preserving the global structure of the input graph.
- We include explicit features of the gene expression profile in the Node Information/Attribute matrix.
- We apply the proposed method on different datasets and obtain higher performance compared to the state-of-the-art approaches.
- We have developed an open-source Github project for the proposed pipeline.

## MATERIALS AND METHODS

### Datasets

The datasets used in this study are publicly available, annotated scRNA-seq data from human and mouse pancreas tissue, drawn from the NCBI’s Gene Expression Omnibus with accession number GSE84133 [20]. The datasets were generated by following the inDrop method under Illumina HiSeq 2500 to determine the transcriptomes of over 12 000 individual pancreatic cells from four human donors and two mice strains. Table 1 depicts the details of datasets including tissue, accession number, number of cells and number of genes. To evaluate and generalize the performance of the proposed method, we have performed experiments on six different datasets listed on Table 1.

**Table 1:** Details of the datasets used in this work including tissue, accession number, number of cell types, number of cells and number of genes

Dataset	Tissue	Accession	No. of cells	No. of genes
Baron-Human1 (BHuman1)	Human-Pancreas	GSM2230757	1,937	20,125
Baron-Human2 (BHuman2)	Human-Pancreas	GSM2230758	1,724	20,125
Baron-Human3 (BHuman3)	Human-Pancreas	GSM2230759	3,605	20,125
Baron-Human4 (BHuman4)	Human-Pancreas	GSM2230760	1,303	20,125
Baron-Mouse1 (BMouse1)	Mouse-Pancreas	GSM2230761	822	14,878
Baron-Mouse2 (BMouse2)	Mouse-Pancreas	GSM2230762	1,064	14,878

### Proposed method

Given complex, high-dimensional scRNA-seq data, we aim to predict cell–cell interactions by creating a pipeline that analyzes single-cell data and converts it to a graph format, performing the prediction using GNNs. We consider the gene expression profile from scRNA-seq data and convert it to an undirected attributed graph, $G$, in which cells and cell–cell interactions are represented by nodes and edges respectively. More formally, given an undirected attributed graph $G$= $(V,E,\mathbf{X})$, where $V$ is a finite set of nodes (cells), $E$ is a finite set of edges (cell–cell interactions), in which $e_{ij}$ = $(v_{i}, v_{j}) \in E$ and $x_{v_{i}}$ is the attribute vector associated with the node $v_{i} \in V$. Also, $A$ = $(a_{ij})_{N \times N}$ represents the adjacency matrix of graph $G$, where $a_{ij}$ = $1$ if $e_{ij} \in E$ and $a_{ij}$ = $0$ otherwise, and $N$ is the number of nodes. We aim to predict the likelihood of a connection between $v_{i}$ and $v_{j}$.

Our proposed method consists of three main steps: (1) preprocessing step (Figure S1.a), (2) CCN construction (Figure S1.b) and (3) applying the GCN (Figure S1.c). Before applying the GCN, the primary step is to preprocess the data (Section 2.2.1). Once the data are preprocessed, a CCN is constructed using SoptSC (Section 2.2.2) in Step 2 (Figure S1.b). The last module of the pipeline uses GCN for prediction. This is the main step of our framework that consists of four main phases before feeding a GCN: (1) feature (gene) selection in the pooling layer; (2) subgraph extraction; (3) node information matrix construction; and (4) Deep Graph Convolutional Neural Network (DGCNN) learning. All these phases are explained in the next few sections.

#### Step1: data preprocessing

To any scRNA-seq data downstream analysis, a critical step is to preprocess the data to reduce the effects of noise in the samples. To this end, we followed a standard preprocessing pipeline in scRNA-seq data analysis [21]. This step includes basic filtering, normalization, log transformation, and scaling, as shown in the first step of the pipeline depicted in Figure S1.a. Low-quality cells would hamper the downstream analysis. These cells may have been damaged or dead during the sequencing process, and are represented by the low number of expressed genes. Based on the pipeline [21], cells with less than 200 expressed genes, and genes expressed in less than three cells have been filtered out. For example, in BHuman1, we filtered out 5387 low-expressed genes that were detected in less than three cells and kept 14 739 genes. We further investigated the distribution of the data (Figure S3), as a data-specific quality-control step to filter low-quality cells. The number of genes expressed in the count matrix is typically between 500 and 4000 genes, with a dense distribution of the number of expressed genes over the total count per cell for less than 4000 genes. As such, we filtered out seven cells to remove low-quality ones. This step is performed to remove low-quality cells and poorly expressed genes.

Normalization is performed to balance the data by bringing it to a common scale without changing any values or losing any information. The top genes in the dataset are visualized before and after normalization in Figure S4.a and S4.b, respectively. The Counts Per Million normalization method is used to normalize the data. Once normalization is performed, data matrices are $log(x+1)$ transformed. After per-gene quantification, we selected a subset of highly variable genes to use in downstream analyses as they are informative of the variability in the data. To achieve this, we chose a commonly used technique in [22] and defined the set of highly variable genes given a normalized dispersion higher than 0.5 after normalization, yielding 2546 genes. For preprocessing, we used Scanpy [23], a specifically designed package to analyze scRNA-seq datasets.

#### Step2: CCN construction

SoptSC (Similarity-matrix based optimization for single-cell data analysis) successfully performs multiple inference tasks such as unsupervised clustering, pseudo-temporal ordering, lineage inference and marker gene identification based on a cell–cell similarity matrix. The cell–cell similarity matrix $S$ is learned from the original scRNA-seq data matrix, i.e. gene expression matrix $X$ of size $m \times n$ with $m$ genes and $n$ cells, using a low-rank representation model [24]. The element $S_{ij}$ (=$S_{ji}$) of similarity matrix $S$ measures the degree of similarity between cell $i$ and cell $j$ [8]. Also, a CCC graph $G$ is constructed using adjacency matrix $A$, which is derived from similarity matrix $S$, where $A_{ij} = 1$ if $S_{ij}> 0$, or $A_{ij} = 0$ otherwise. In this work, we constructed the CCN using this method.

#### Step 3.1: gene selection in pooling layer

Downsampling is crucial in graph analysis, which is included in the pooling layer of our method framework. The pooling layer consists of selecting genes (with a threshold $\tau $) by Information Gain (IG) feature selection. This step provides the node attribute information (side information) of each node, i.e. explicit features. IG, as a feature selection method, computes the reduction in entropy by splitting the dataset based on a given value of a random variable and measures how important or relevant the feature is. This is done by estimating the information gained from each variable and choosing the one with the maximum value. Based on Equation (1), the largest information gain is equal to the smallest entropy. IG is calculated by subtracting the weighted entropy values from the original entropy values by the following Equation (2). In other words, IG measures how changes to the dataset affect the distribution of the classes or target variables: 


(1) \begin{align*}& H(X) = -\sum p(X)\log p(X) \,,\end{align*}

where, for dataset $\mathbf{X=}\{x_{i}\}$, $H(X)$ is the probability of randomly picking an element of the class. 


(2) \begin{align*}& I(X,a)= H(X)-H(X|a)\,,\end{align*}

where $\mathit{I(X,a)}$ represents the information gain in dataset $\mathbf{X=}\{x_{i}\}$ for variable $a$, $H(X)$ is the original entropy of $X$ and $H(X|a)$ is the conditional entropy for the given variable $a$.

#### Step 3.2: enclosing subgraph extraction

The subgraph induced from the network by the union of $u$ and $v$’s neighbours up to $k-$ hops is called the enclosing subgraph for a node pair ($u$, $v$). The hop is the distance that node features can traverse in one hop. Enclosing subgraph extraction involves extracting the local enclosing subgraphs around the target nodes $u$ and $v$. The enclosing subgraph is extracted from the training data, which contains both positive (existent) and negative (non-existent) sets of sampled links, based on $h$-hop neighbours for the target nodes $u$ and $v$. Figure S5 depicts an example of the $1$-hop enclosing subgraphs for target nodes $(A,B)$ and $(C,D)$.

#### Step 3.3: node information matrix construction

In the node information matrix, each row corresponds to the node’s feature vector, which is represented as $X$. In the SEAL [15] approach, there are three components in the node information matrix:

**Structural information: node labeling** Nodes are labeled based on their structural roles using a graph labelling method, the Double-Radius Node Labelling algorithm (explained in [15]). The main purpose of this step is to label every node in the enclosing subgraph to distinguish the target nodes between which a link should be expected. Later, in the DGCNN learning step, nodes will be sorted in a sort pooling layer, based on their structural roles, indicated by node labels. Labels are assigned to nodes in such a way that the target nodes $u$ and $v$ are labelled 1. Also, the radius of node $i$ with respect to target nodes, namely $(d(i,u), d(i,v)$, can be used to define its position. Thus, nodes on the same orbit are given the same label. In other words, larger labels are assigned to nodes that have a larger radius (farther nodes) with respect to target nodes. This algorithm can be better understood by following the diagram of Figure S6, which satisfies the following conditions:

1. if $d(i, x) + d(i, y) \neq d(j, x) + d(j, y),$ then $d(i, x) + d(i, y) < d(j, x) + d(j, y) \Leftrightarrow f_{l}(i) < f_{l}(j);$
2. if $d(i, x) + d(i, y) = d(j, x) + d(j, y),$ then $d(i, x) d(i, y) < d(j, x)d(j, y) \Leftrightarrow f_{l}(i) < f_{l}(j).$

where $f_{l}(i)$ is the label assigned to node $i$ and $(d(i,x), d(i,y))$ is the double radius.

**Latent information: node embedding** The node embedding methods, i.e. Node2Vec [17], LINE [25] and Spectral Clustering (SC) (explained in Section 3.1), give the feature representation of nodes in a graph. Thus, an additional step is required to learn the features of the edges from node embedding to predict links as a binary classification problem.

**The Negative injection** trick, as explained in [15], is used to help to generate the node embeddings. This consists of adding the negative (non-existent) set of sampled links, $E_{n}$, to the positive (existent) set of sampled links, $E$, and generating the embeddings on $G^{\prime} = (V, E \cup E_{n})$. The node embedding method used in our method is Node2Vec [17].

**Explicit information: node attributes** Both latent and explicit features of each node are included in the node information matrix $X$ for its corresponding row in $X$. Latent feature learning methods learn low-dimensional latent representation or embedding for each node using matrix factorization [26]. The adjacency or Laplacian matrix derived from the graph can be used for matrix factorization. On the other hand, the explicit features contain side information about the individual nodes, available in the form of Node Attributes. The SEAL method [15] shows significant performance improvement when combining both latent and explicit features.

#### Step 3.4: learning DFCNN

DGCNN [27] is a deep learning architecture that is designed to operate on graph data. DGCNN is divided into three main parts:

1) Graph Convolution layers: localized graph convolutions are used to extract the hidden feature information of nodes from the graph. DGCNN consists of multiple convolutional layers in which the output of each convolutional layer is passed to a hyperbolic tangent ($tanh$) non-activation function. A GCN with four convolutional layers is shown in Figure S7.

2) SortPooling Llayer: in the SortPooling layer, the unordered node attributes of the graph from the spatial graph convolutions layer are fed as the input. The main purpose of this layer is to sort the feature descriptors, each of which represents a node. Rather than summing up these node features, the SortPooling layer arranges them in a consistent order and outputs a sorted graph representation with a given size. Then, it can be read and trained by standard convolutional neural networks. Nodes are sorted using graph labelling methods, based on their structural roles, in descending order using the last layer’s output, $Z^{h}$. Once the nodes are sorted, the next step is to unify the sizes of the output tensor. The main intention behind it is to unify the graph sizes to $k$ by deleting the last $n-k$ rows if $n>k$, or adding $k-n$ zero rows otherwise. The output of the SortPooling Layer is shown in Figure S8.

3) Traditional Convolutional and Dense Layers: these layers are used to make a prediction based on the sorted graph representations generated by the SortPooling layer. The architecture of DGCNN is shown in Figure S9. Given the adjacency matrix $A \in{\{1,0\}}^{n \times n}$ of graph $G$ with $n$ number of nodes and each node containing the $c$ dimensional feature vector as well as the node information matrix $X \in R^{n \times c}$ of an enclosing subgraph with each row representing the node, DGCNN employs the following convolution layer: 


(3) \begin{align*}& Z = f(\tilde{D^{-}1}\tilde{A}XW) \,,\end{align*}

where $\tilde{A} = A + I$, $I$ is the identity matrix, $\tilde{D}$ is the diagonal degree matrix with $\tilde{D_{i,i}} = \sum{j} \tilde{A_{i,j}}$, $W$ is a trainable graph convolutional parameters, $f$ is a non-linear activation function and $Z \in R^{n \times c^{\prime}}$ is the output activation matrix.

The graph convolution incorporates each node’s hidden representation by aggregating attribute information from its neighbours. The graph convolution can be split into four different steps:

1. Linear feature transformation is applied to the node information matrix $X$, by multiplying it by $W$.
2. Node information is propagated to neighbouring nodes as well as the node itself by $\tilde{A}XW$.
3. Each row is normalized by multiplying it by $D^{-1}$.
4. A non-linear activation function is applied to obtain the output.

## PERFORMANCE EVALUATION

We conduct extensive experiments to evaluate the performance of our proposed method. We included seven methods for comparison: four state-of-the-art latent feature learning methods (Section 3.1) including Node2Vec [17], LINE [25], Deepwalk [16] and SC, as well as WLNM [19], GAE [28] and VGAE [29] (Section 3.2).

We use the binary operators (proposed in Node2Vec) in our evaluation process. These operators over the corresponding feature vectors of nodes $u$ and $v$, i.e. $f(u)$ and $f(v)$, are utilized to generate the edge/link embedding $g(u,v)$ for edge $e = (u,v)$:

- **Average:** 

(4) \begin{align*}& fx(u)\boxplus f(v) = \frac{f(u)+ f(v)}{2}\,.\end{align*}

- **Hadamard: ** 

(5) \begin{align*}& f(u) \boxdot f(v) = f(u) * f(v) \,.\end{align*}

- **Weighted-L1:** 

(6) \begin{align*}& ||f(u) \cdot f(v)||_{{\overline{1}}} = |f(u) - f(v)| \,.\end{align*}

- **Weighted-L2:** 

(7) \begin{align*}& ||f(u) \cdot f(v)||_{\overline{2}} = |f(u) - f(v)|^{2} \,.\end{align*}

The original code for Node2Vec, Deepwalk and LINE is used in our comparison study. The node embeddings generated from these methods are used to generate the link embeddings (as explained in Section 3.1). Then, we used logistic regression as the classifier to predict the links. To evaluate the performance with the other methods including GAE, VGAE ([19], we used the default settings. We used different hyperparameters for each method as described in Table S2. To implement the core of our method, we used the base implementation of the SEAL method. Then, to evaluate the performance of the results, we used 90–10% of data as training and testing sets respectively. To generate FPR/TPR distribution of the datasets, we have taken the mean of the corresponding values.

We used Area Under Curve (AUC), accuracy, precision, recall, F1-score and receiver operating characteristic curve (ROC curve) as evaluation metrics. To calculate the evaluation metrics, we used training and testing data which consisted of both positive (existent) and negative (non-existent) links. As a negative set, we randomly chose an equal number of unconnected pairs of nodes from the network with no edge connection between them. We arbitrarily remove 10% of links as testing data and the remaining 90% are used as training data. The statistical information of the network extracted from datasets (discussed in Section 2.1) is shown in Table S1.

### Latent feature methods

Given a network $G$ with a finite set of nodes (or vertices) $V$ and a finite set of edges $E$, latent features are the features in the low dimensional representations of nodes $V$ computed using matrix factorization [26]. The matrix can be the adjacency matrix or the Laplacian matrix derived from the network $G$. Node2Vec [17], LINE [25] and DeepWalk [16] are examples of network/node embedding algorithms that we use as latent feature methods to learn latent features. In [30], these network embedding methods were found to implicitly factorize some network matrix representation. These methods are summarized as follows:

(1) Node2Vec: the Node2Vec [17] model for latent feature learning is an application of the Word2Vec paradigm. The latter is a framework for word embedding used to learn continuous feature representations of nodes in networks using the skip-gram model. The goal is to optimize a neighbourhood-preserving likelihood objective to learn these representations. As an extension of the skip-gram architecture of networks, Node2Vec is an embedding approach that works on neighbour nodes and generates low-dimensional embedding by converting graph/network into numerical representations. A second-order random walk approach is used to generate the numerical representation of the nodes in the graph. The idea behind Node2Vec is to use flexible, biased random walks that can trade-off between local and global network views. This approach returns feature representations that maximize the likelihood of preserving network neighbourhoods of nodes in a $d$-dimensional feature space [17].

(2) DeepWalk: DeepWalk [16] learns $d$- dimensional latent feature representations using local information obtained from uniform random walks. To capture network topology information, Deepwalk introduced an unsupervised strategy that learns features that capture the graph structure independently of the labels’ distribution, rather than mixing the label space as part of the feature space [16].

(3) LINE: LINE [25] is a network embedding model designed for embedding very large-scale information networks, which contain millions of nodes and billions of edges. This method generates low-level embeddings by preserving both the first- and second-order proximity of nodes. Furthermore, this method incorporates a novel edge-sampling technique that improves the efficiency of the model [25].

(4) SC: SC is a matrix factorization [26] technique that performs an eigendecomposition of a graph $G$, more specifically, the normalized Laplacian matrix $L$, and takes top $k$ eigenvectors as the feature representation of nodes, i.e. node embedding vectors, $Z$. The edge score is calculated as the sigmoid function, $Z \times Z^{T}$.

### Graph-based methods

(1) WLNM: WLNM is a subgraph-based link prediction method that extracts the enclosing subgraphs around the target nodes to learn graph structure features for link prediction. The number of nodes in the subgraph is denoted by the user-defined integer $K$. The Palette-WL algorithm, a variant of WL that is fast and order-preserving, is used to label nodes. The enclosing subgraph is then represented as an adjacency matrix by WLNM. A fully connected neural network is trained on these adjacency matrices, together with their labels, to learn the existence of links. WLNM has three steps: (a) enclosing subgraph extraction, (b) subgraph pattern encoding and (c) neural network training.

2) SEAL): SEAL framework for link prediction learns general graph structure features from local subgraphs rather than complete networks. The method takes as input the enclosing subgraphs around the links and returns the likelihood that the links exist. SEAL consists of three steps: (a) enclosing subgraph extraction, (b) node information matrix creation and (c) GNN learning. The default GNN used in SEAL is DGCNN [Section 2.2.6] [15].

## RESULTS AND DISCUSSION

Overall, compared to other methods, our method achieves improvement in performance in terms of AUC. Table 2 shows the performance (AUC) of our method and latent methods. For all the datasets, Node2Vec outperformed all other approaches for three of the four operators. It means Node2Vec excels in generating low-dimensional embeddings of nodes in networks and achieving a neighbourhood-preserving objective. Thus, we chose Node2Vec as the node embedding method in our framework.

**Table 2:** Comparison of our method with latent methods in terms of performance (AUC)

Operator	Method	BHuman1	BHuman2	BHuman3	BHuman4	BMouse1	BMouse2
Average	Node2Vec	0.4999	0.5162	0.5035	0.5177	0.5049	0.5127
	LINE	0.5061	0.5034	0.5053	0.4968	0.5142	0.5093
	DeepWalk	0.5029	0.5052	0.5	0.5148	0.5099	0.513
	SC	0.4728	0.5464	0.5361	0.531	0.5043	0.5312
Hadamard	Node2Vec	0.9748	0.9766	0.9833	0.9711	0.9564	0.9726
	LINE	0.7077	0.7908	0.5696	0.8279	0.8474	0.8494
	DeepWalk	0.956	0.9634	0.9514	0.9615	0.9558	0.9635
	SC	0.9392	0.9625	0.9501	0.9623	0.9589	0.9648
Weighted L1	Node2Vec	0.9887	0.9885	0.9917	0.9851	0.9798	0.9846
	LINE	0.7204	0.7474	0.5528	0.8421	0.894	0.8848
	DeepWalk	0.9867	0.9857	0.9859	0.982	0.9813	0.9812
	SC	0.9743	0.9757	0.9696	0.9716	0.969	0.9694
Weighted L2	Node2Vec	0.9896	0.9895	0.9919	0.9862	0.9802	0.9846
	LINE	0.7243	0.7474	0.5603	0.8487	0.8989	0.8865
	DeepWalk	0.9869	0.9866	0.9857	0.9825	0.9823	0.9822
	SC	0.9748	0.9752	0.9687	0.9736	0.9752	0.9729
	SEGCECO	**0.9985**	**0.9980**	**0.9989**	**0.9982**	**0.9975**	**0.9972**

Table 3 depicts the performance (AUC) of our method with other GNN-based methods, such as GAE, VGAE, WLNM. Among them, our method performs best with approximately 99% AUC. We anticipate that the improved performance of our method is due to the proposed pooling layer in the framework, which uses IG as the feature selection method to select the top $\tau $ attributes (i.e. genes) as explicit features in the node information matrix, $X$, resulting in better prediction. 

**Table 3:** Comparison of our method with other GNN-based methods in terms of performance (AUC)

Datset	GAE	VGAE	WLNM	SEGCECO
BHuman1	0.9835	0.9852	0.9832	**0.9985**
BHuman2	0.9859	0.9805	0.9839	**0.9980**
BHuman3	0.9876	0.9869	0.9889	**0.9989**
BHuman4	0.9838	0.9764	0.9773	**0.9982**
BMouse1	0.9841	0.9764	0.9673	**0.9975**
BMouse2	0.9838	0.9829	0.9744	**0.9972**

Moreover, Figure S10 plots the ROC curve for DeepWalk, Node2Vec, LINE, SC, GAE, VGAE, WLNM and our method on the BHuman1 dataset. It is noticeable that our method surpasses other approaches since the curve is closer to the top-left corner, indicating better performance.

Robust inferences are essential to minimize false discoveries and help reduce the number of validations to perform, which is especially useful when experiments are expensive.

Here, positive means interacting cells and negative means non-interacting cells. Observing Figure S11 reveals that our method obtained the lowest FPR of 0.0135 among all the approaches, implying that there is a very low probability that our method will predict non-interacting cells as interacting cells. This, in other words, means when the cells do not have interactions, the chances of inaccurate predictions, i.e. the cells interact, are minimal. Furthermore, our method performs best in predicting actual interactions, that is, when there exist interactions between cells, the method predicts the same. The same behaviour is detected in other datasets as well. The ROC curves, FPR and TPR distribution on other datasets can be found in the Supplementary Material (Figures S12–S21). Thus, it can be concluded that our method yields the best results for all datasets when it regards distinguishing between interacting and non-interacting cells and making predictions.

Moreover, Accuracy, Precision, Recall and F1-score are the commonly used evaluation metrics to illustrate the performance of the model. Recall evaluates the model for correctly identifying CCC. Precision shows the percentage of predictions accurately made by the model. Table 4 shows the AUC, accuracy, precision, recall and F1-score of link prediction using our method framework on different datasets. Our method shows a performance of around 99% for all measures, indicating that our model can accurately predict cell–cell interactions and discriminate interacting cells from non-interacting cells. Based on the findings of the above-mentioned comparison, we conclude that our method surpassed all other approaches with 99% AUC, accuracy and other performance measures across all datasets.

**Table 4:** Performance metrics (AUC, Accuracy, Precision, Recall and F1-score) of our method for the datasets

Datset	AUC	Accuracy	Precision	Recall	F1-score
BHuman1	0.9985	0.9928	0.9872	0.9987	0.9929
BHuman2	0.9980	0.9903	0.9915	0.9891	0.9903
BHuman3	0.9989	0.9923	0.9925	0.9921	0.9923
BHuman4	0.9982	0.9886	0.9862	0.9913	0.9887
BMouse1	0.9975	0.9854	0.9800	0.9908	0.9854
BMouse2	0.9972	0.9878	0.9800	0.9954	0.9876

Although the model can be used to infer cell–cell interactions, the generated predictions are still hypotheses to be validated experimentally. Transcriptomics may not fully represent a biologically accurate view of CCC, as mRNA and protein abundance may be uncoupled by post-transcriptional and post-translational processes [31]. To improve the predictive models, one possible approach is through multi-omics integration. Borrowing information from other omics technologies can improve confidence in the results. Novel techniques and methods such as Mass Cytometry and spatial transcriptomics (ST) allow researchers to analyze the transcriptome of tissues in a more real context, with the addition of information about proteins. Also, the limited scale of experiments focusing on the examination of ligand and receptor pairs within specific cell lines or tissues has been addressed by the emergence of advanced techniques in ST. This valuable information facilitates broader analyses of extracellular interactions on a larger scale.

Also, we can indeed improve SEGCECO, and other computational methods for cell–cell interaction prediction, by addressing preprocessing to reduce the noise in the dataset. Metacells are defined as small and cohesive groups of cells that are highly similar to each other, derived from single-cell sequencing data, that represent resampling of the same cellular states. They are used to simplify the analysis of large scRNA-seq datasets. The use of metacells often improves the results of downstream analyses including visualization, clustering, differential expression, cell-type annotation, gene correlation, imputation, RNA velocity and data integration while reducing sampling variance, resulting in a more robust estimate of the mean gene expression level for a given cell state compared to individual cells. Applying an algorithm, Metacell-2. [32], or an algorithm similar or inspired by that algorithm, to our model in the preprocessing step could help improve results in downstream analysis and could potentially provide information for filtering parameters. This algorithm directly controls metacell sizes, and implements a new adaptive outlier detection module and a rare-gene-module detector allowing very high sensitivity for detecting transcriptional states that are present in as little as 0.01% of data (citation). This integration of metacells and visualization of how metacell graphs are partitioned could help us refine our filtering parameters to exclude outlier cells that are doublets and include rare cells that would have otherwise been filtered.

In terms of the main step of cell–cell interaction prediction, a study has shown that integrating scRNA-seq data with ST data can help in the evaluation of cell–cell interaction methods. This integration allows the characterization of cell–cell interactions into short-range and long-range interactions using spatial distance distributions between ligands and receptors. Therefore, by using metacells to simplify the scRNA-seq data and integrate it with ST data, it is possible to improve the prediction of cell–cell interactions. However, the specific improvement would depend on the exact methods and datasets used. We will explore more how incorporating metacells into SEGCECO’s operation could potentially enhance its performance in dealing with the complexities of single-cell transcriptomics data.

## BIOLOGICAL ASSESSMENT

To interpret the network inferred, information such as gene ontology or pathway and other metadata associated with the cells in the dataset gain insight into functional relationships between cells. For example, a set of genes involved in a particular pathway are highly co-expressed across different cells in the network. This could indicate a functional relationship between those cells related to that particular pathway. There are several network analysis toolboxes and resources that can be used to validate the predicted cell–cell interactions. Also, these tools can identify highly connected nodes or subnetworks, which may represent key regulators or effectors of the functional relationships between cells.

GeneMANIA Cytoscape’s plugin has been used in this assessment since it provides an interactive network visualization of the predicted functional relationships between the query genes (genes of interest) and other genes based on co-expression, co-localization and other data sources.

Also, the ReactomeFIViz app helps to find pathways and network patterns related to diseases by accessing Reactome pathways and the Functional Interaction network. Functional Interaction (FI) network is a highly reliable, manually curated pathway-based protein functional interaction network covering over 60% human proteins, and allows the construction of an FI sub-network based on a set of genes.

We ran the ReactomeFIViz to find underlying sub-networks in the query list. The results represent and validate some examples of interactions that GeneMANIA found based on the input. As shown in Figure S22, the visualization of the networks, there are four sub-networks related to the BHuman1 dataset. Query genes that are indicated with purple, are grouped in four separate clusters. Genes involved in the first cluster were analyzed using Reactome and six out of seven identifiers in the sample were found, where 64 pathways were hit by at least one of them. We listed the important ones in Table S1. For example, in the Other interleukin signalling pathway, interleukins are low molecular weight proteins that bind to cell surface receptors and act in an autocrine and/or paracrine fashion. They were first identified as factors produced by leukocytes but are now known to be produced by many other cells throughout the body. They have pleiotropic effects on cells that bind them, impacting processes such as tissue growth and repair, hematopoietic homeostasis, and multiple levels of the host defence against pathogens where they are an essential part of the immune system [33]. Also, in the Cytokine Signaling in Immune system pathway, cytokines are small proteins that regulate and mediate immunity, inflammation, and hematopoiesis. They are secreted in response to immune stimuli, and usually act briefly, locally, at very low concentrations. Cytokines bind to specific membrane receptors, which then signal the cell via second messengers, to regulate cellular activity [34]. Additionally, we ran GeneMANIA on the input list of the top 20 genes, extracted from the pooling layer which indicates the most effective genes in the predicted interactions among cells. Figure S23 represents the key regulators or effectors of the functional relationships between cells in separate network types, such as co-expression, predicted, shared protein domain and other factors.

## CONCLUSION

In this paper, we propose a pipeline for performing cell–cell interaction prediction in scRNA-seq data using GCN and an attributed graph. This article demonstrates how scRNA-seq data in the form of a gene expression matrix is transformed into a graph representation, i.e. a CCN, to predict cell–cell interactions in scRNA-seq datasets.

Our proposed method works with undirected, attributed graphs created from the gene expression profiles of the individual cells. The architecture of our method includes a pooling layer that coarsens the graph attributes from the scRNA-seq data while preserving the global structure of the input graph. The pooling layer is followed by the enclosing subgraph extraction, node information matrix construction and, finally, GCN that convolves over the graph to encode the representation of both local and global attributes. The experimental results have shown that our proposed method outperforms previous state-of-the-art techniques. We evaluated our method using AUC, accuracy, precision, recall and F1-score evaluation metrics. Findings show a performance of approximately 99% for all performance measures across all the datasets. We empirically proved that our method yields better results in terms of AUC relative to the previously proposed latent and subgraph-based methods. Thus, we conclude that our method outperforms other approaches in predicting cell–cell predictions and distinguishing interacting from non-interacting cells.

Our proposed method also opens up new research opportunities to work with networks in which there is a special structure such as heterogeneous CCN, networks with explicit domain features for nodes and edges, and directed or multi-modal graphs. In addition to the application of cell–cell link prediction, the proposed method could be applied to node classification, node clustering, graph partitioning and graph classification. We would also foresee applying our method to domains such as disease–gene or drug–target associations, knowledge graph completion and recommendation systems, among others.

Also, to enhance the accuracy of cell–cell interaction prediction models like SEGCECO, addressing preprocessing steps to minimize dataset noise is crucial. Utilizing metacells, cohesive groups of highly similar cells obtained through resampling, during preprocessing can lead to improved outcomes in various downstream analyses. Additionally, integrating metacells with ST data allows for a more nuanced evaluation of short-range and long-range cell–cell interactions, potentially enhancing the performance of SEGCECO in handling the intricacies of single-cell transcriptomics data.

**Key Points**

- We introduce a statistically significant pooling layer that employs information gain as an approach for coarsening graph attributes from the scRNA-seq data while preserving the global structure of the input graph.
- We include explicit and implicit features from the gene expression profile in the Node Information/Attribute matrix using subgraph embedding.
- The proposed method utilizes an attributed graph convolutional neural network to predict cell-cell communication from single-cell RNA-seq data, capturing both latent and explicit attributes of the cells’ gene expression profiles.
- SEGCECO tackles the challenge of transforming high-dimensional and sparse single-cell RNA-seq data into a graphical form via the construction of a cell–cell communication network from a similarity matrix derived from gene expression data.
- Performance evaluations on human and mouse pancreas tissue datasets demonstrate that SEGCECO surpasses both latent feature-based models and state-of-the-art network-based methods in link prediction.

## Supplementary Material

SupplementaryMaterial_SEGCECO_bbae160

## Data Availability

The original datasets used in this article were drawn from the NCBI’s Gene Expression Omnibus, as discussed in Section 2.1. The processed data are available in GitHub Repository], at https://github.com/sheenahora/SEGCECO/tree/master/data.

## Author Biographies

**Akram Vasighizaker** received her PhD in Computer Science and is continuing her research with the Biomedical Science Department at the University of Windsor. Her main research interests include single-cell data analysis, machine learning and cancer research.

**Sheena Hora** received her MSc in computer science from the University of Windsor in 2022. She is currently working with Amazon as a Software Development Engineer. Her research interests include deep learning and machine learning.

**Raymond Zeng** received his BSc in molecular genetics and microbiology from the University of Toronto in 2020. He is currently a bachelor’s student in computer science at the University of Windsor. His research interest includes bioinformatics, machine learning and cancer molecular genetics.

**Luis Rueda** is a full professor in computer science at the University of Windsor. His main research focuses on machine learning in spatial transcriptomics and cancer biomarkers. He is a senior member of the IEEE and associate editor of IEEE TCBB and NetMAHIB.
