# Supplementary material for: SEGCECO: Subgraph Embedding of Gene expression matrix for prediction of CEll-cell COmmunication
Source: Brief Bioinform. 2024 Apr 11;25(3):bbae160. doi: 10.1093/bib/bbae160 (PMC11009470; doi:10.1093/bib/bbae160)
Supplement: SupplementaryMaterial_SEGCECO_bbae160 [file supplementarymaterial_segceco_bbae160.docx]

**Supplementary Materials for**

SEGCECO: **S**ubgraph **E**mbedding of **G**ene expression matrix for prediction of **CE**ll-cell **CO**mmunication

Akram Vasighizaker, Sheena Hora, Raymond Zeng, and Luis Rueda

1. Preprocessing step of the proposed framework.

1. Cell-cell network construction step of the proposed framework.

1. Main algorithm step of the proposed framework.

Figure S1: Pipeline of the proposed framework for prediction of cell-cell communication.

Figure S2: $k$−hop proximity of target node marked in red and the neighbors of the target node in the $k$ −hop neighborhood within $k$ = 1, 2, and 3.

Figure S3: Distribution of the data (BHuman1).

|  |  |
| --- | --- |
| (a): Before normalization. | (b): After normalization. |
|  |  |

Figure S4: Highly variable genes before and after normalization (BHuman1).

Figure S5: 1-hop enclosing subgraphs for target nodes (A, B) and (C, D).

Figure S6: Node labeling approach.

Figure S7: A GCN with four convolutional layers.

Figure S8: Overview of the Sort Pooling layer’s output.

Figure S9: Overview of DGCNN architecture.


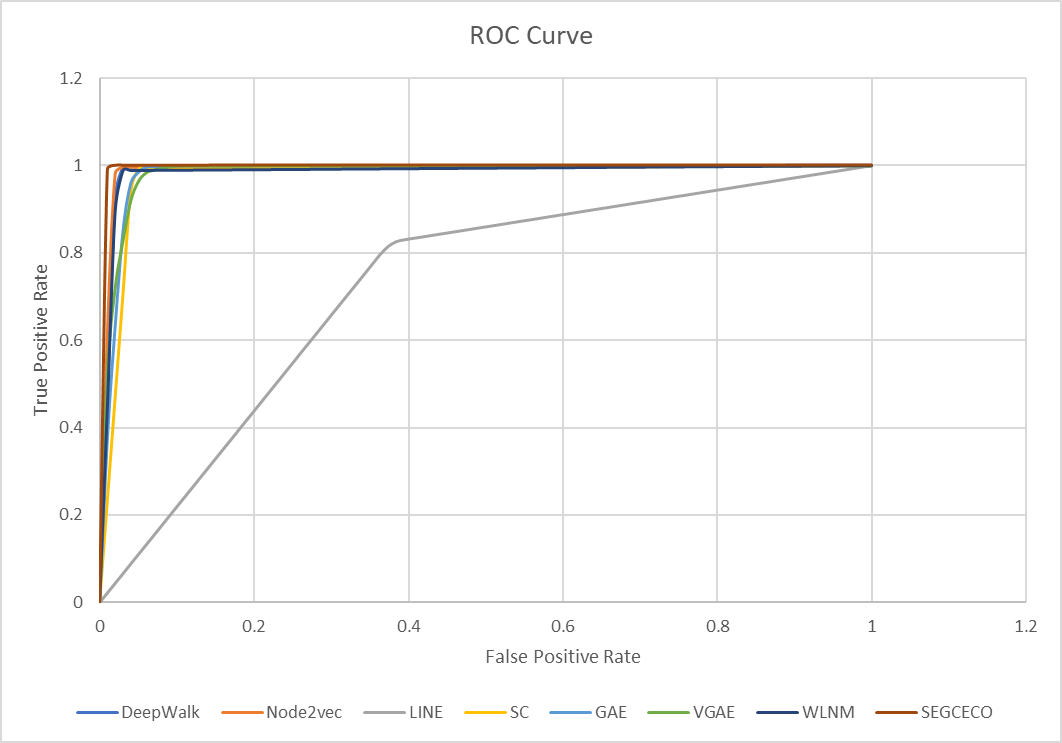


Figure S10: ROC Curve for BHuman1 dataset.


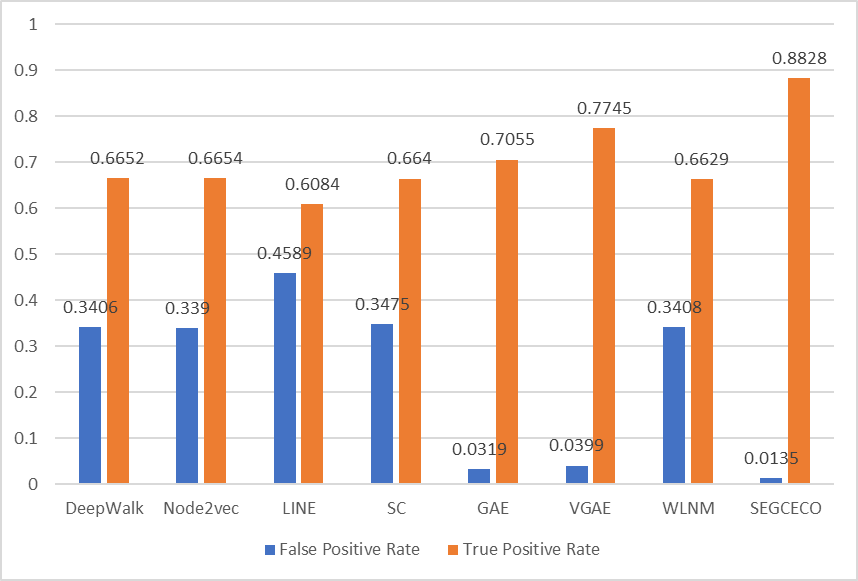


Figure S11: False Positive Rate and True Positive Rate distribution of BHuman1 dataset.

*
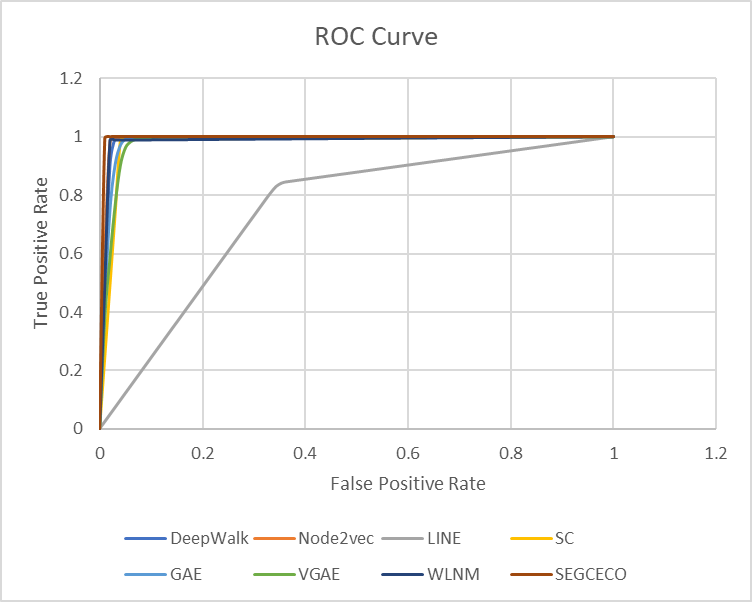
*

Figure S12: ROC Curve for BHuman2 dataset. The curve closer to top-left corner indicates better performance.

Figure 13: False Positive Rate and True Positive Rate distribution of BHuman2 dataset.

*
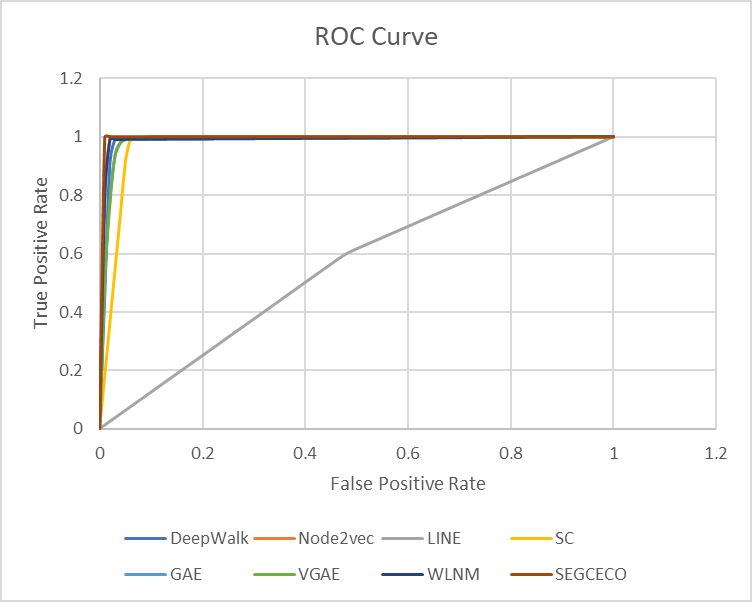
*

Figure 14: ROC Curve for BHuman3 dataset. The curve closer to top-left corner indicates better performance.

Figure S15: False Positive Rate and True Positive Rate distribution of BHuman3 dataset.

*
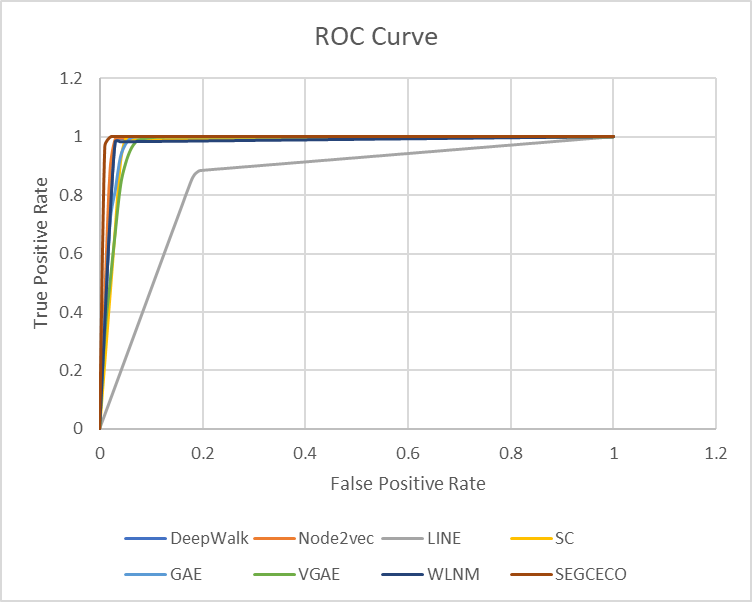
*

Figure S16: ROC Curve for BHuman4 dataset. The curve closer to top-left corner indicates better performance.

Figure S17: False Positive Rate and True Positive Rate distribution of BHuman4 dataset.

*
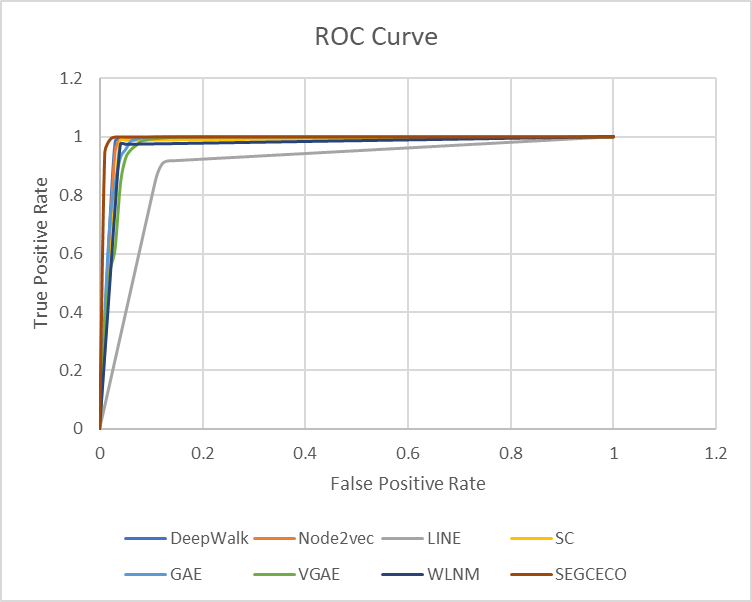
*

Figure S18: ROC Curve for BMouse1 dataset. The curve closer to top-left corner indicates better performance.

Figure S19: False Positive Rate and True Positive Rate distribution of BMouse1 dataset.

*
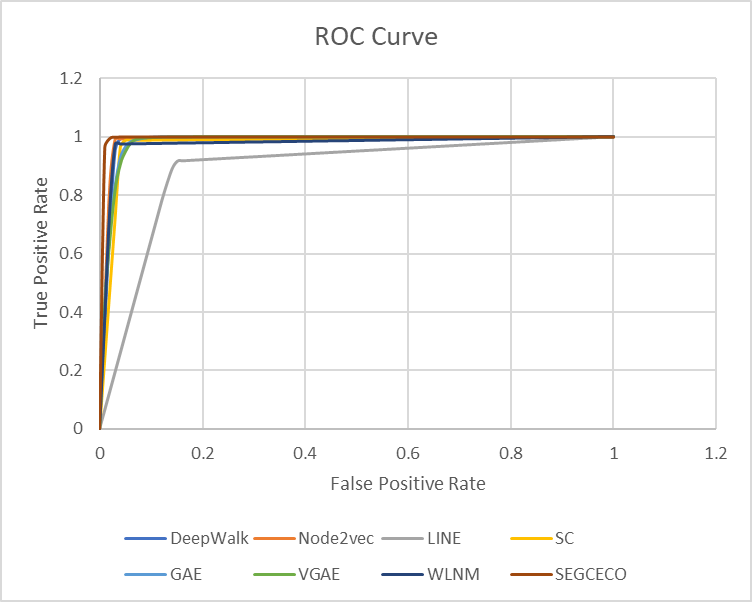
*

Figure S20: ROC Curve for BMouse2 dataset. The curve closer to top-left corner indicates better performance.

Figure S21: False Positive Rate and True Positive Rate distribution of BMouse2 dataset.


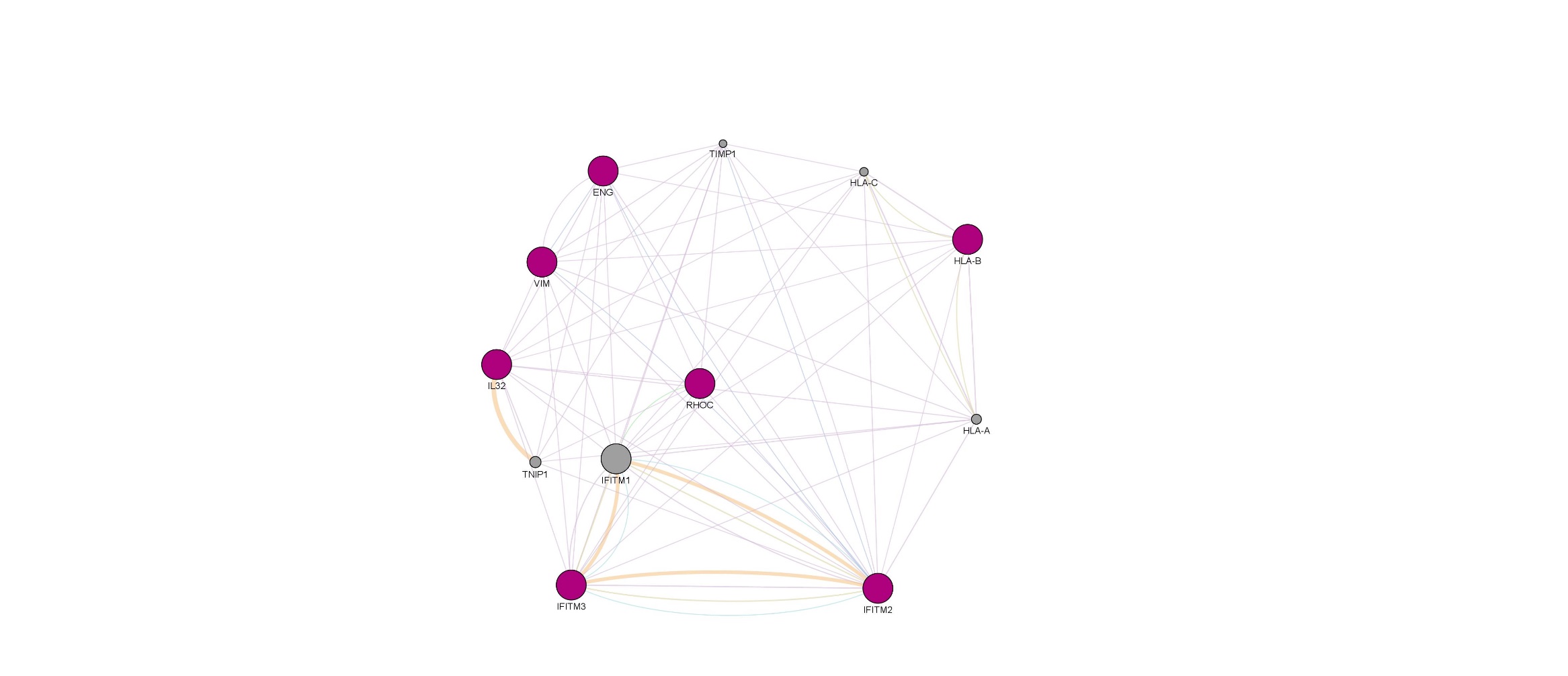


Figure S22: a) Subnetwork 0


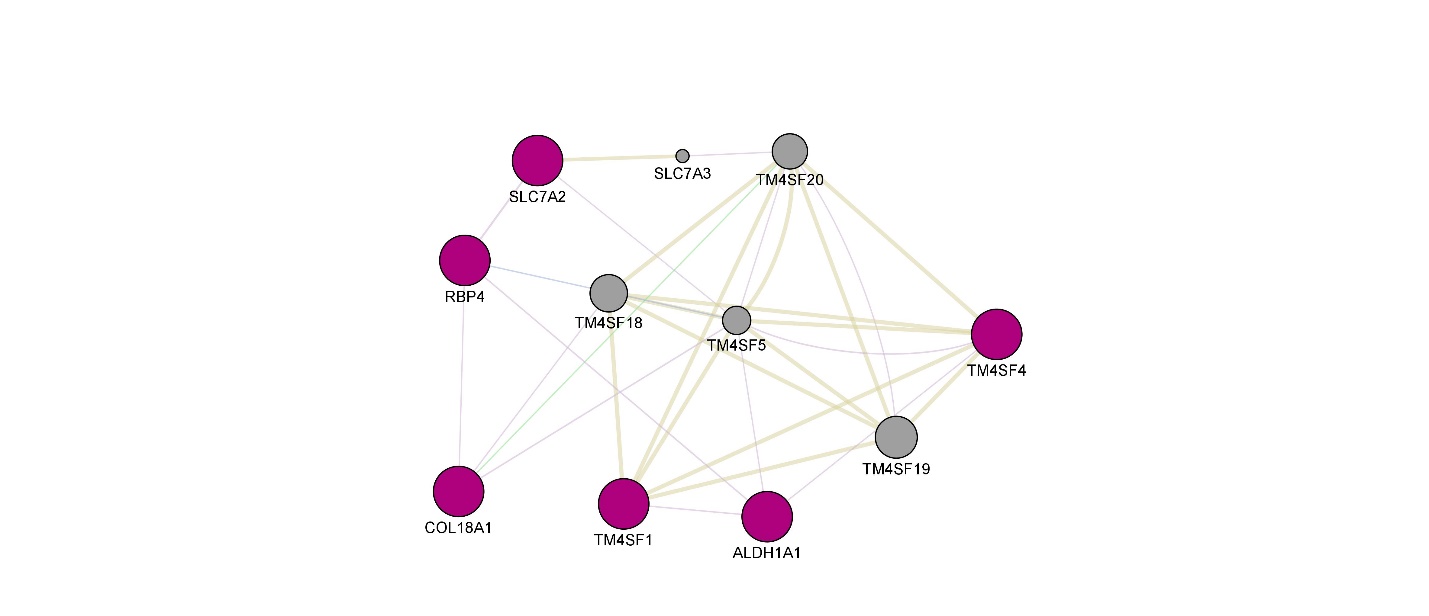


Figure S22: b) Subnetwork 1


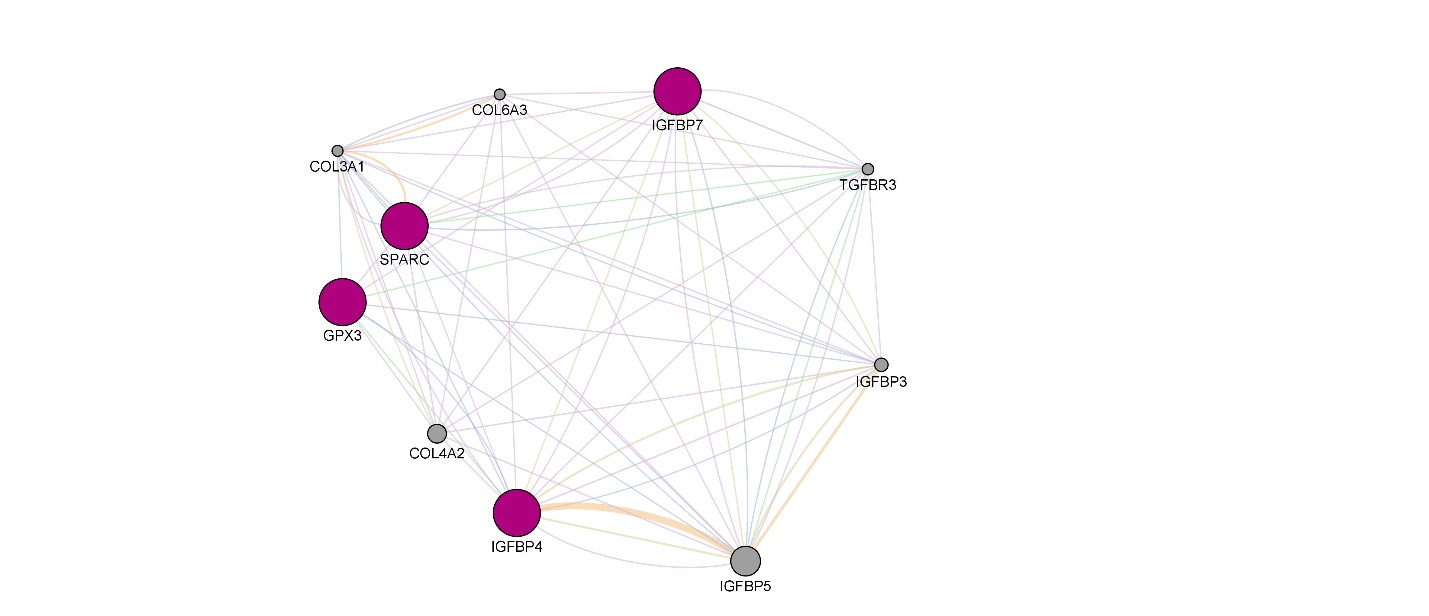


Figure S22: c) Subnetwork 2


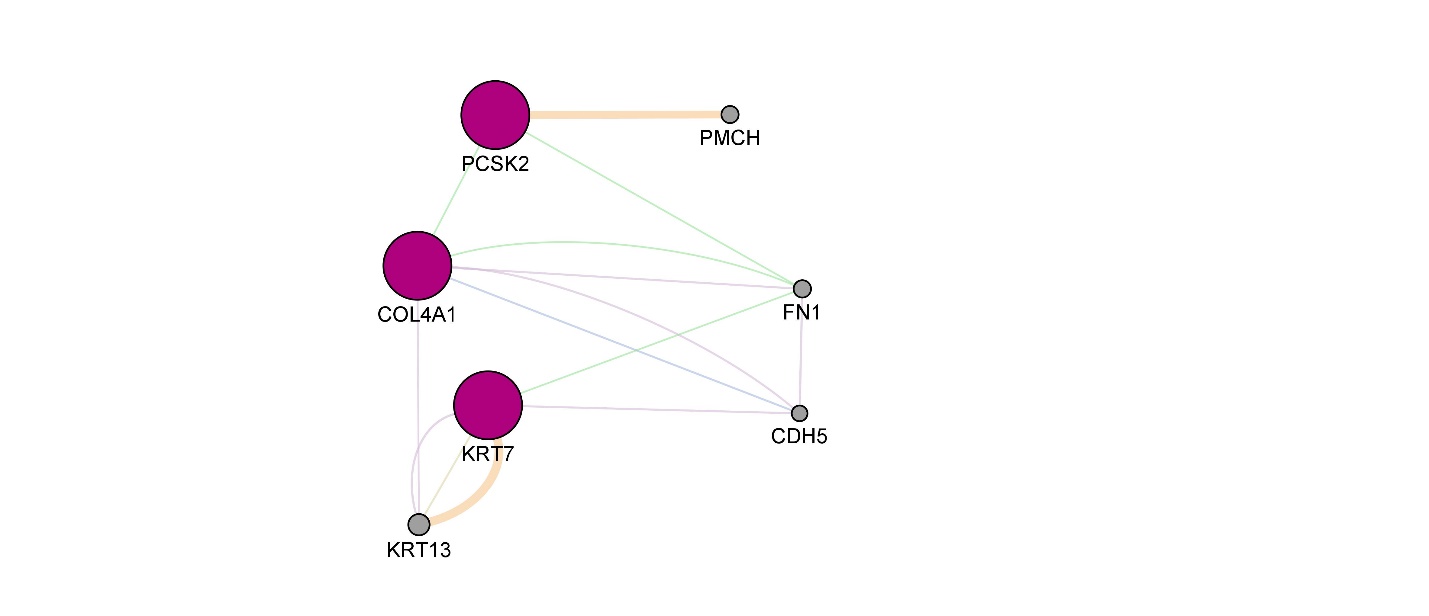


Figure S22: d) Subnetwork 3

Figure S22: Clusters extracted using ReactomeFIVIZ on BHuman1 dataset. The colored nodes indicating the query genes and colored links represent the interactions that connect the nodes (genes). The color coded links showing different interactions. Purple links showing co-expression networks, olive green color indicating shared-protein domain networks, yellow ones are predicted interactions, blue edges link co-localized genes, red one corresponds to physical interactions, light blue showing pathway networks, and light green indicates genetic interactions. Thick links signify the strength of the links, and the size of the nodes highlights the importance of that node in that interaction.

Table S1: The most relevant pathways sorted by p-value in pathway analysis of Bhuman1 dataset

| **Pathway name** | **#Entities found** | **#Entities total** | **Entities pValue** | **Entities FDR** | **#Reactions found** | **#Reactions total** | **Submitted entities found** |
| --- | --- | --- | --- | --- | --- | --- | --- |
| Antigen Presentation: Folding, assembly and peptide loading of class I MHC | 35 | 108 | 1.11E-16 | 3.33E-16 | 13 | 16 | HLA-B |
| Endosomal/Vacuolar pathway | 35 | 82 | 1.11E-16 | 3.33E-16 | 3 | 4 | HLA-B |
| Class I MHC mediated antigen processing & presentation | 35 | 479 | 1.11E-16 | 3.33E-16 | 19 | 48 | HLA-B |
| Antigen processing-Cross presentation | 35 | 195 | 1.11E-16 | 3.33E-16 | 6 | 23 | HLA-B |
| Immunoregulatory interactions between a Lymphoid and a non-Lymphoid cell | 36 | 316 | 1.11E-16 | 3.33E-16 | 5 | 44 | IFITM3;IFITM2;HLA-B |
| Interferon gamma signaling | 36 | 252 | 1.11E-16 | 3.33E-16 | 2 | 18 | HLA-B |
| Adaptive Immune System | 36 | 1012 | 1.11E-16 | 3.33E-16 | 24 | 265 | IFITM3;IFITM2;HLA-B |
| Interferon alpha/beta signaling | 41 | 190 | 1.11E-16 | 3.33E-16 | 2 | 25 | IFITM3;IFITM2;HLA-B |
| SARS-CoV-2 activates/modulates innate and adaptive immune responses | 35 | 227 | 1.11E-16 | 3.33E-16 | 3 | 47 | HLA-B |
| Interferon Signaling | 41 | 397 | 1.11E-16 | 3.33E-16 | 4 | 74 | IFITM3;IFITM2;HLA-B |

| 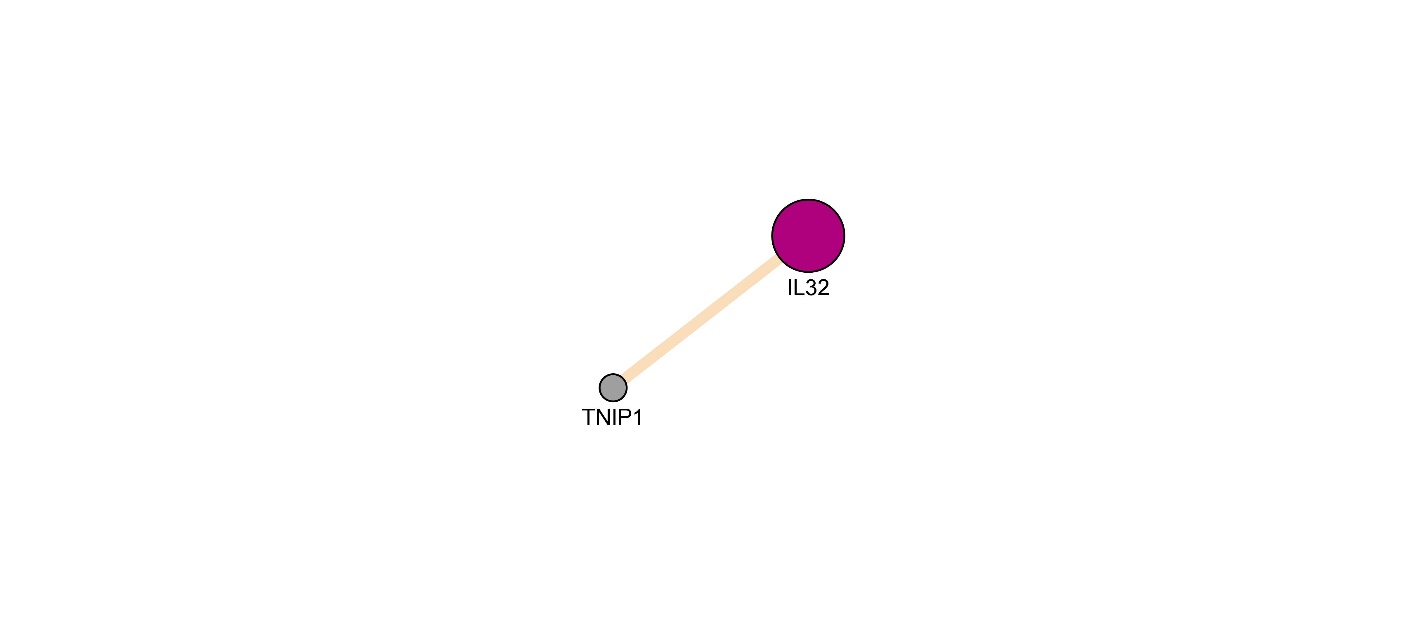 | 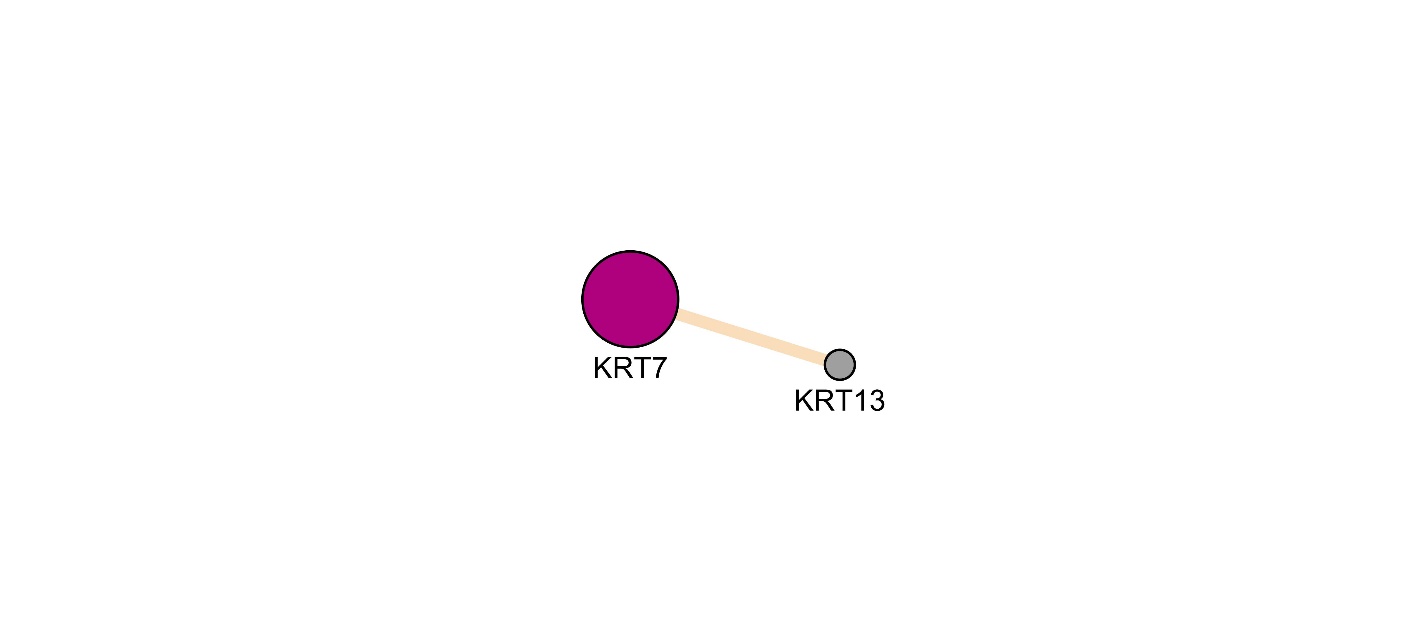 |
| --- | --- |
| 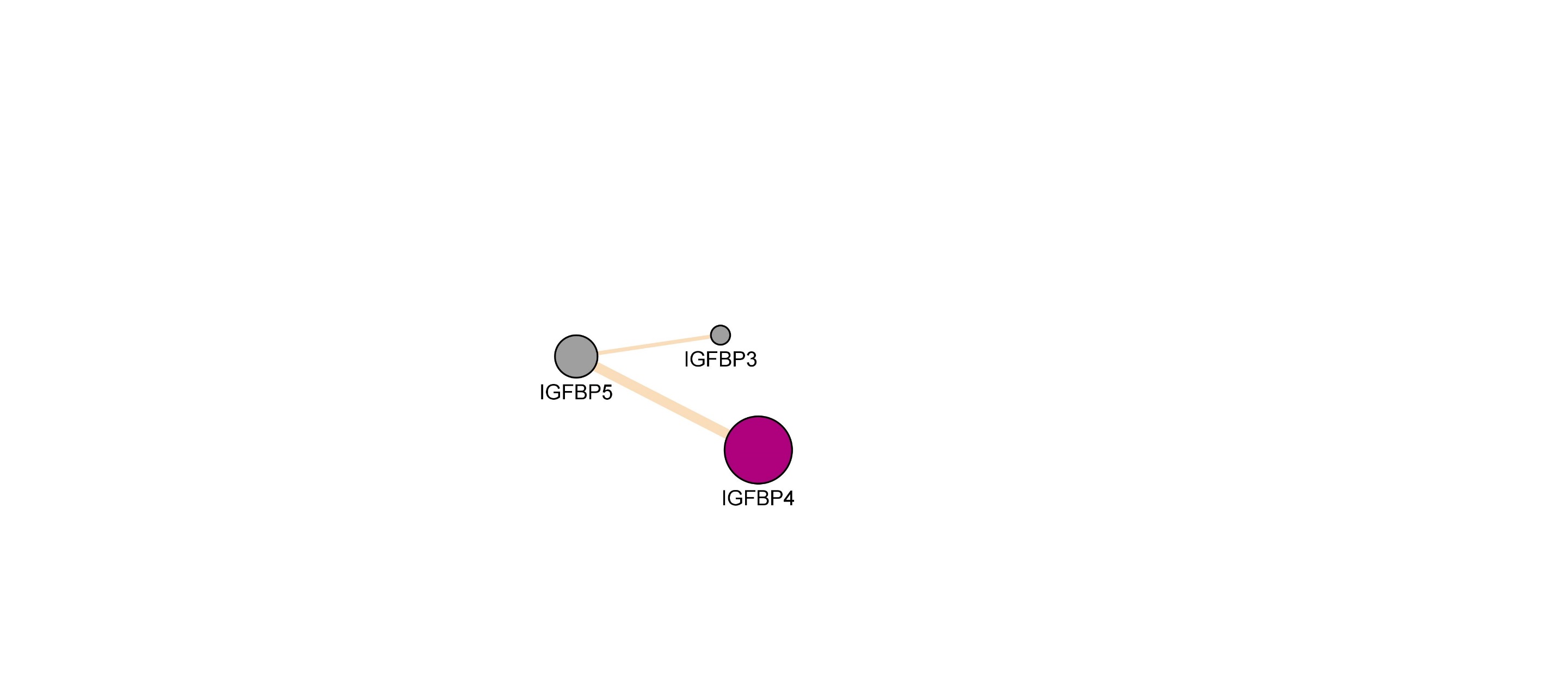 | 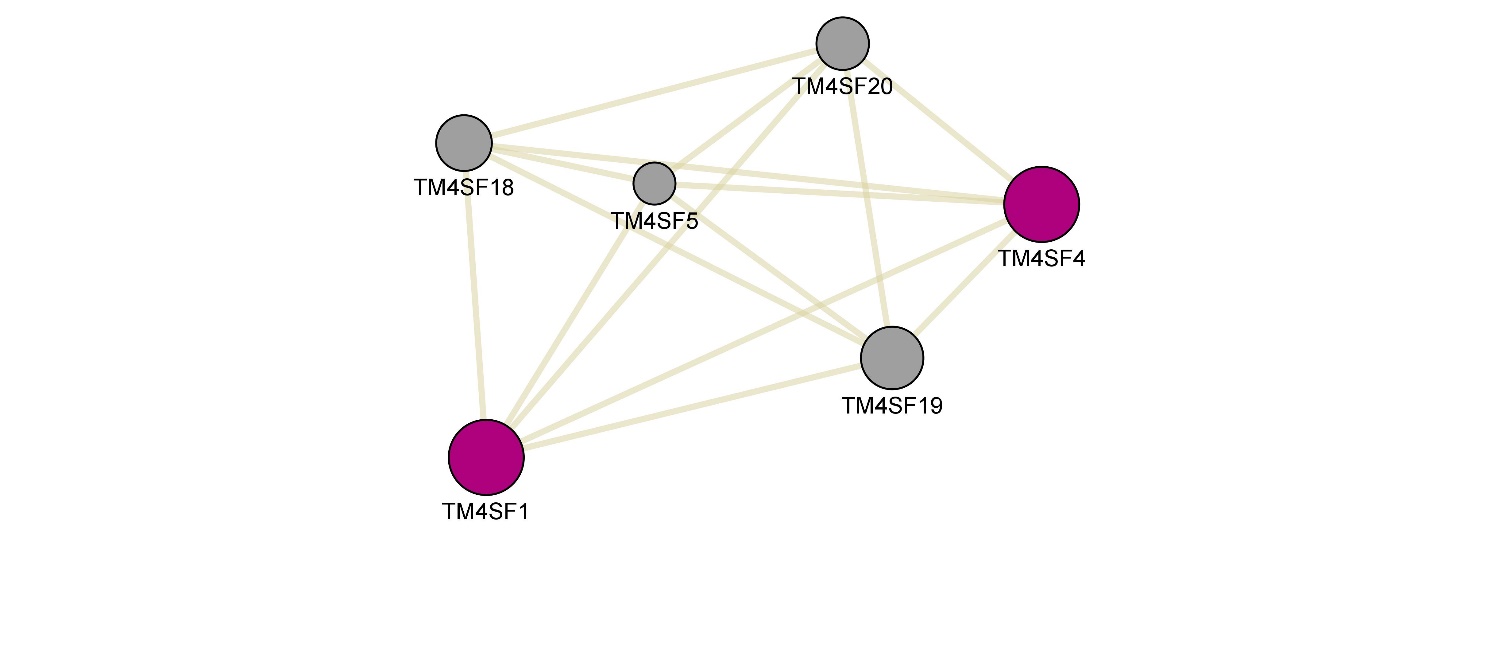 |
| 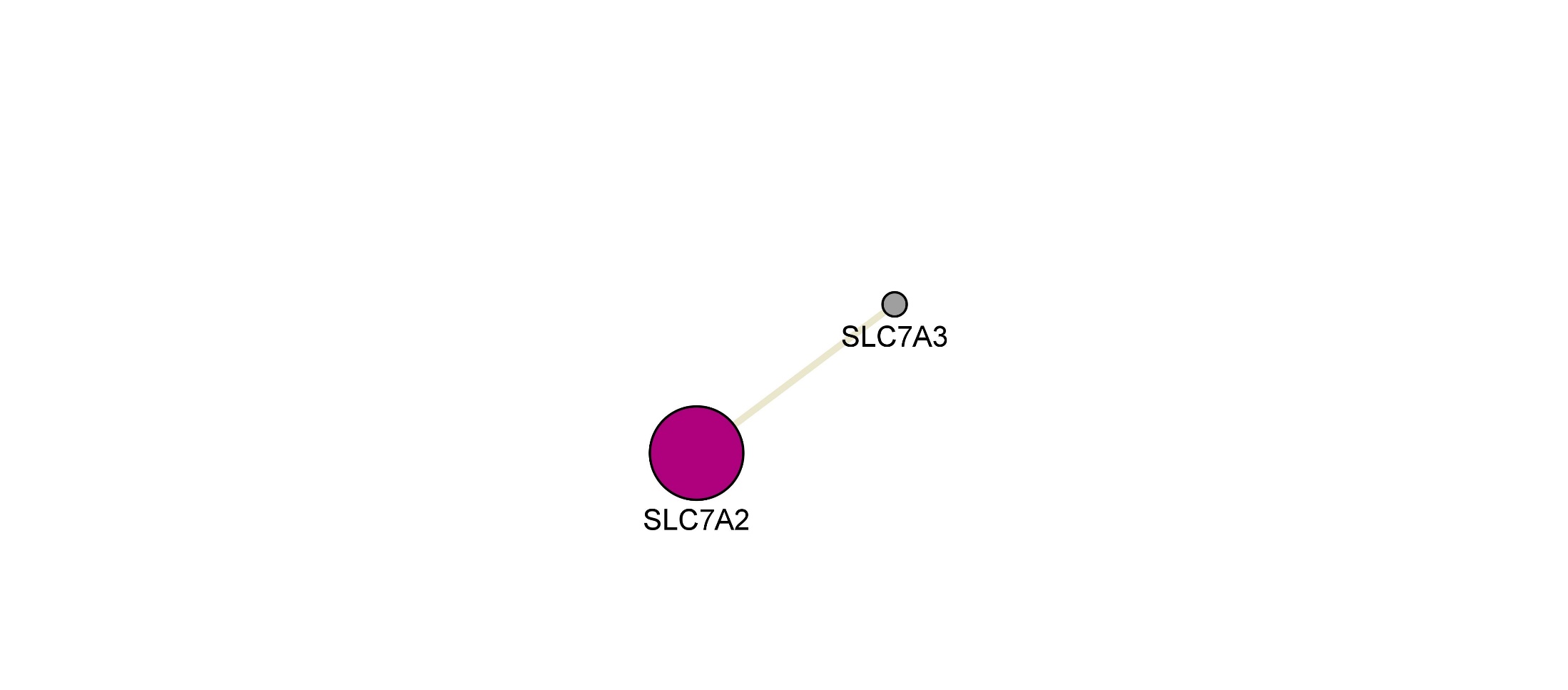 | 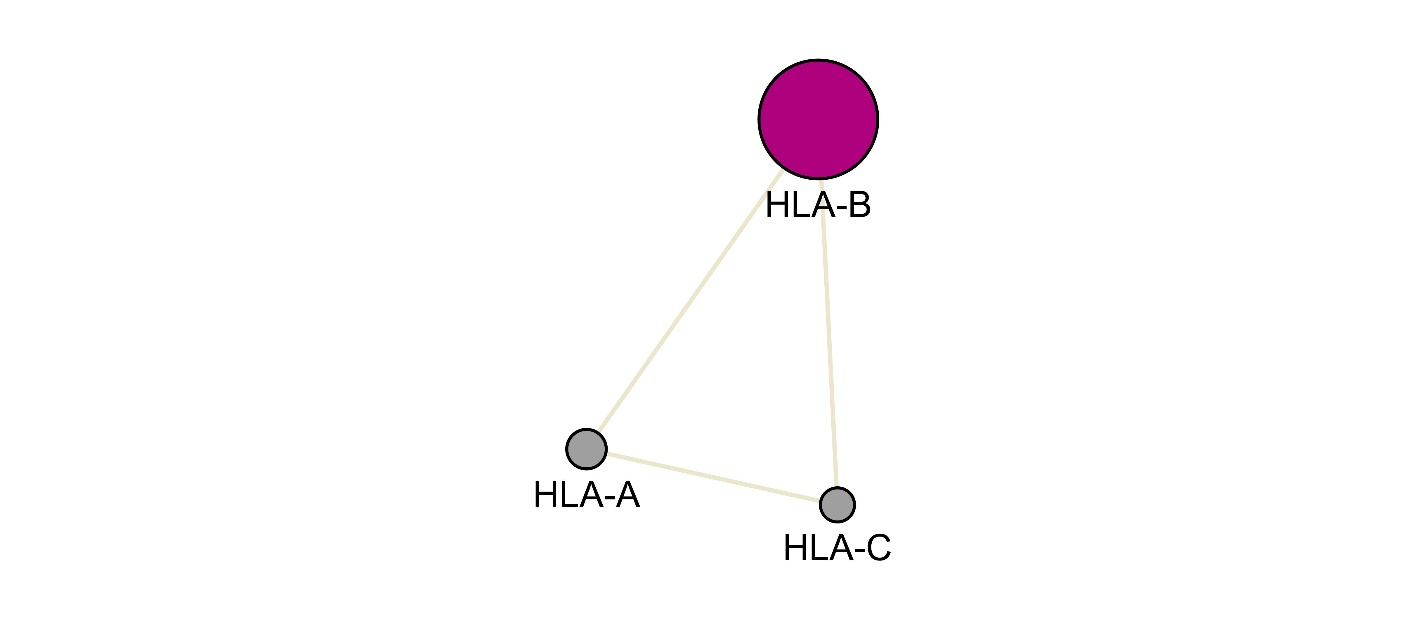 |
| 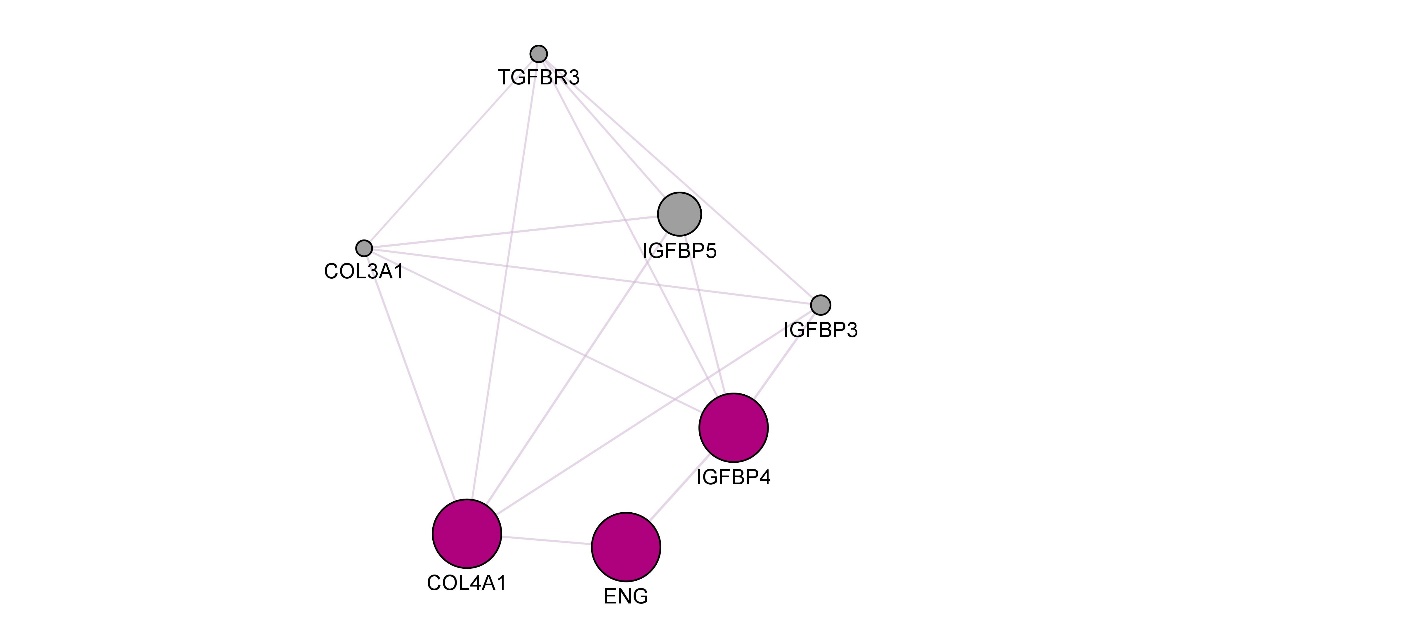 | 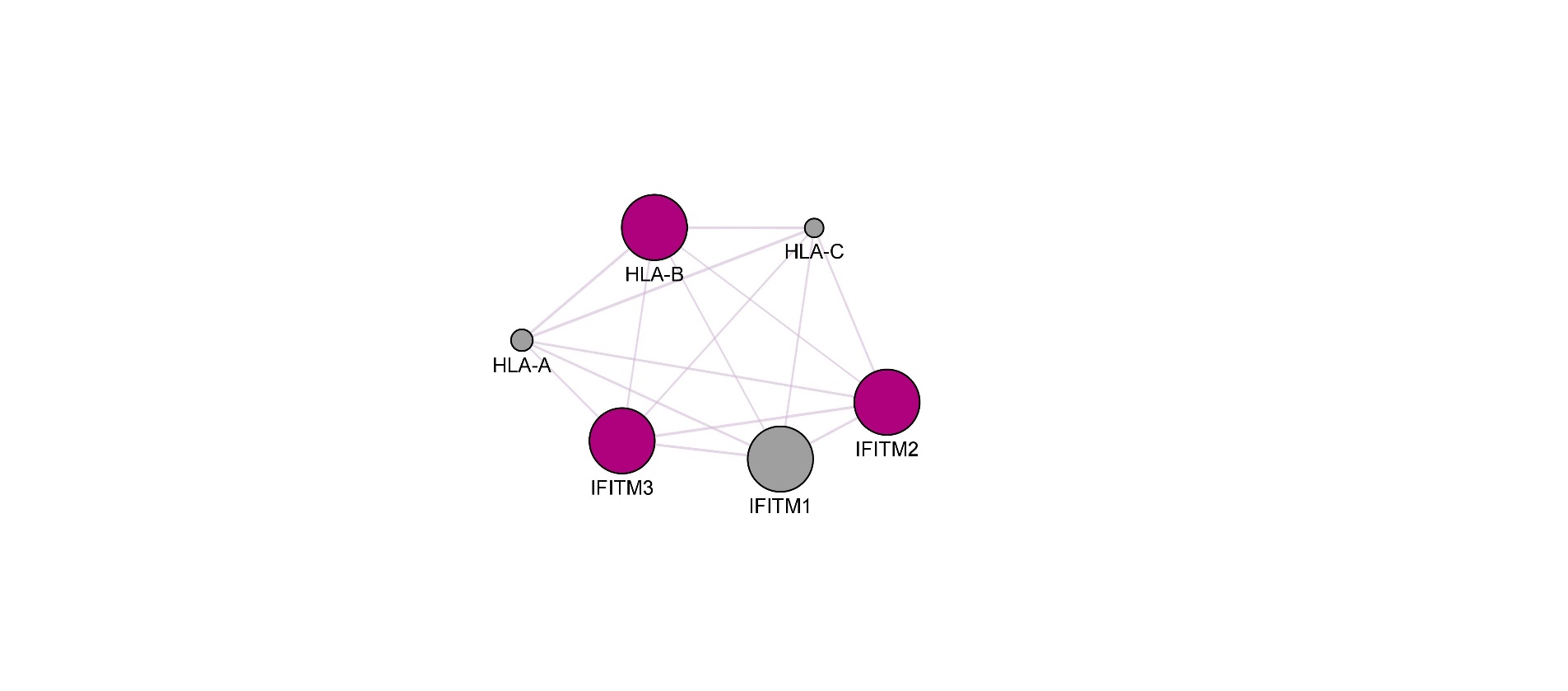 |
| 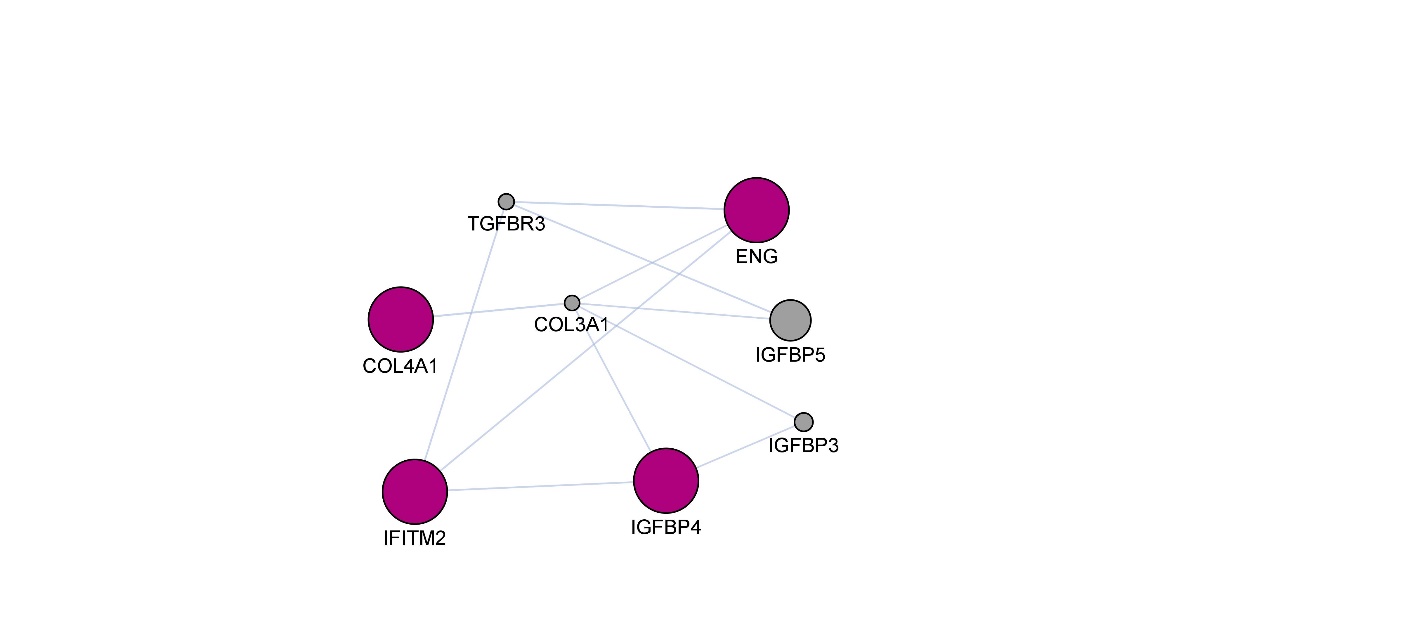 | 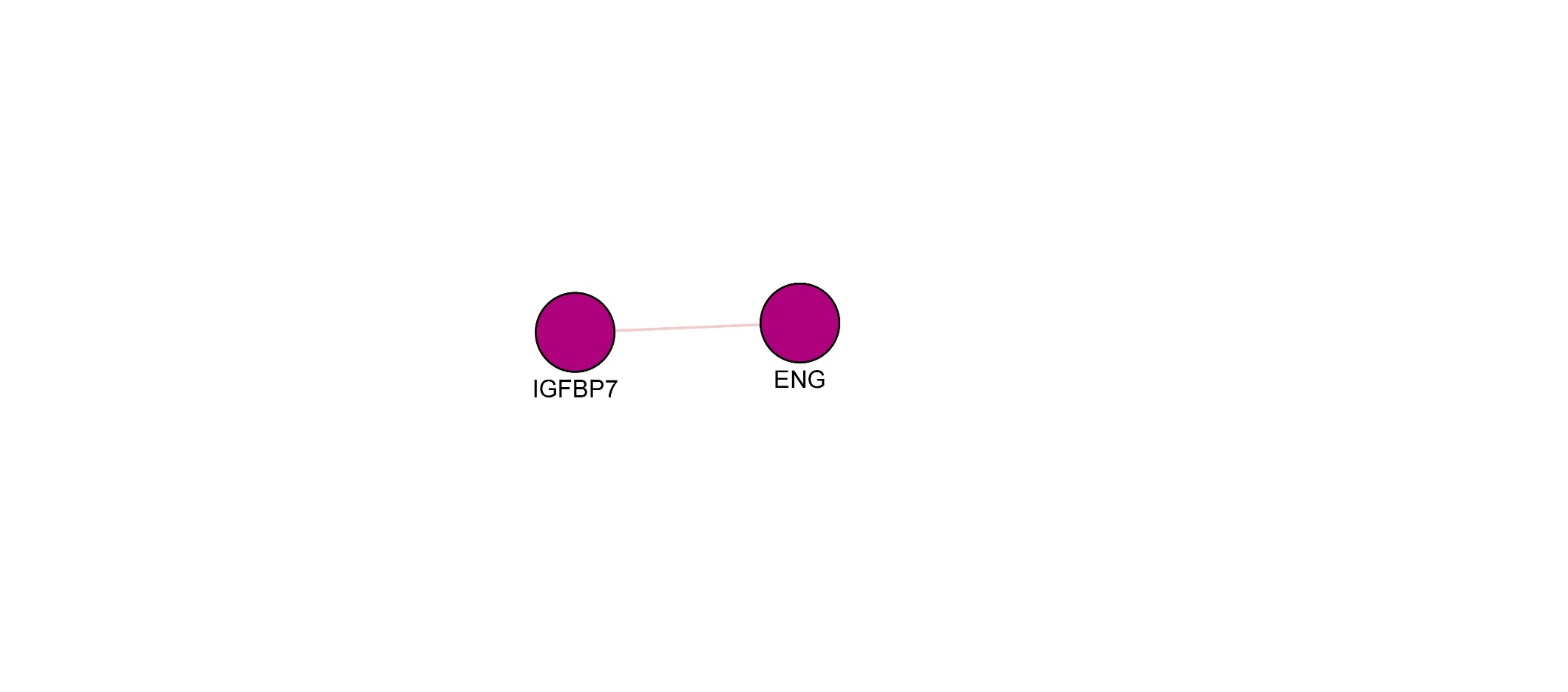 |
| 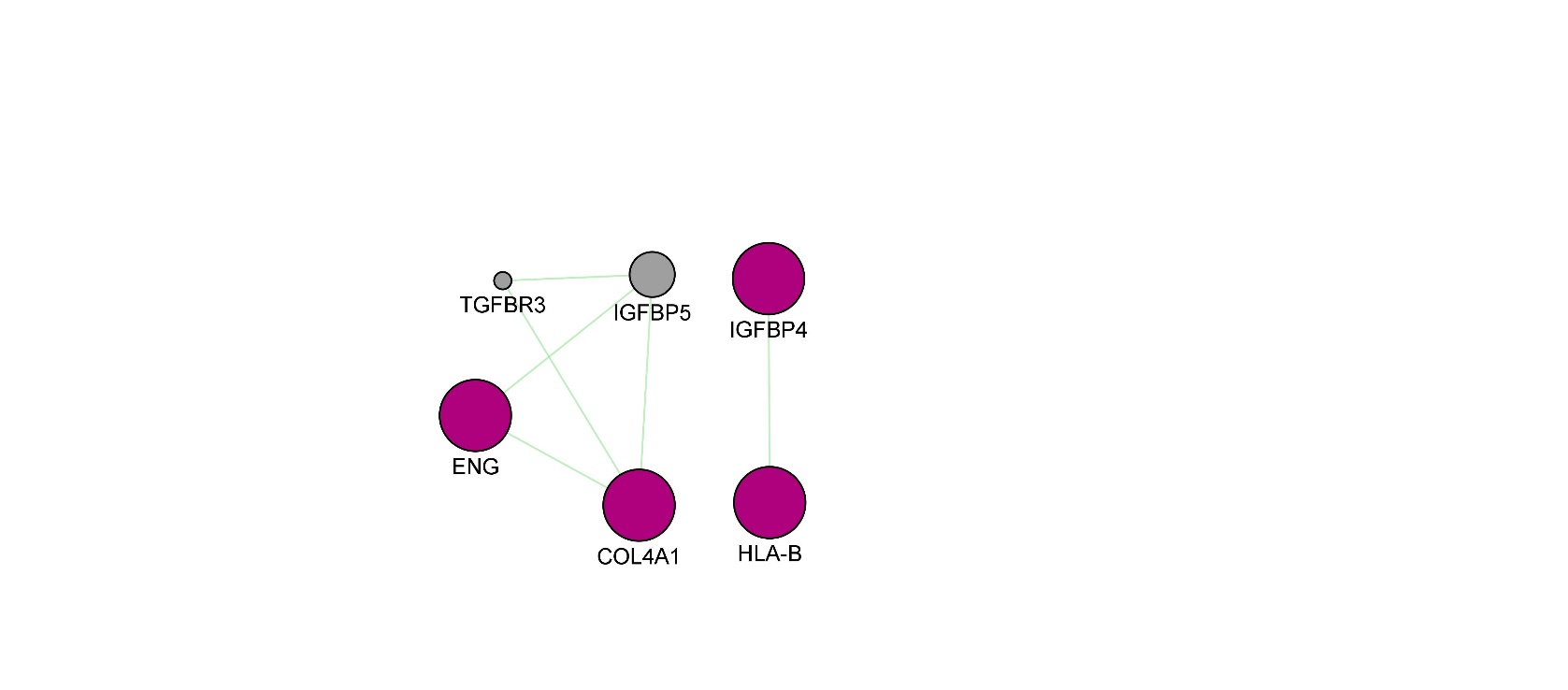 | 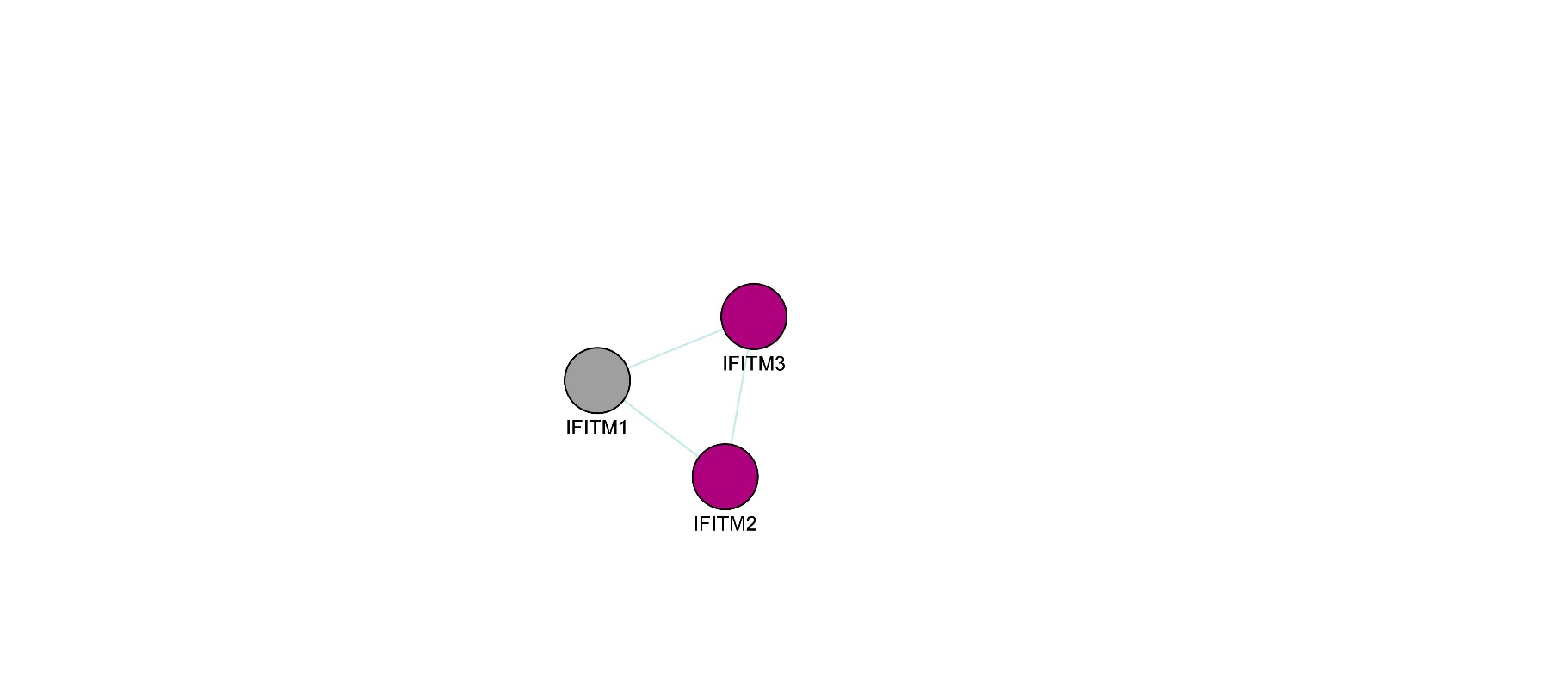 |

Figure S23: sub-networks presenting the key regulators or effectors of the functional relationships between cells in separate network types. Sub-networks are extracted using Genemania on BHuman1 dataset. The colored nodes indicating the query genes and colored links represent the interactions that connect the nodes (genes).The color coded links showing different interactions. Purple links show co-expression networks, olive green colour indicates shared-protein domain networks, yellow ones are predicted interactions, blue edges link co-localized genes, red corresponds to physical interactions, light blue showing pathway networks, and light green indicates genetic interactions. Thick links signify the strength of the links and the size of the nodes highlights the importance of that node in that interaction.
